# SQSTM1/p62 and Mitochondrial Stress in Triple-Negative Breast Cancer: A Critical Pathology Perspective

**DOI:** 10.3390/cancers18213589

**Published:** 2026-11-09

**Authors:** Valeria Zuccalà, Teresa Maria Martorana, Ludovica Pepe, Ida Rizzuto, Giuliana Ciappina, Carmelo Sofia, Maria Adele Marino, Mariamena Arbitrio, Riccardo Cavaliere, Massimiliano Berretta, Giovanni Tuccari, Vincenzo Fiorentino, Antonio Ieni

**Affiliations:** 1 Anatomic Pathology Unit, Department of Human Pathology of Adult and Developmental Age “Gaetano Barresi”, University of Messina, 98125 Messina, Italy; teresa.martorana@studenti.unime.it (T.M.M.); ida.rizzuto@studenti.unime.it (I.R.); giovanni.tuccari@unime.it (G.T.); vincenzo.fiorentino@unime.it (V.F.); aieni@unime.it (A.I.); 2 Department of Biomedical, Dental, Morphological and Functional Imaging Sciences, University of Messina, 98125 Messina, Italy; carm.sofia@tiscali.it (C.S.); mariaadele.marino@unime.it (M.A.M.); 3 Section of Experimental Medicine, Department of Medical Sciences, University of Ferrara, 44121 Ferrara, Italy; giuliana.ciappina@unime.it; 4 Department for the Promotion of Human Science and Quality of Life, San Raffaele Roma University, 00166 Rome, Italy; mariamena.arbitrio@uniroma5.it; 5 Clinical Pathology Unit, Department of Human Pathology of Adult and Developmental Age “Gaetano Barresi”, University of Messina, 98125 Messina, Italy; riccardo.cavaliere@unime.it; 6 Department of Clinical and Experimental Medicine, University of Messina, 98125 Messina, Italy; massimiliano.berretta@unime.it

**Keywords:** triple-negative breast cancer, SQSTM1/p62, immunohistochemistry, mitochondrial quality control, mitophagy, tissue biomarkers, neoadjuvant therapy

## Abstract

Sequestosome 1 (SQSTM1/p62) links autophagy to cellular stress responses and can be assessed by immunohistochemistry. In triple-negative breast cancer (TNBC), however, its staining has no single biological interpretation. This selective narrative review examines human tissue studies alongside experimental evidence, focusing on the pathological assessment of mitochondrial stress. Human evidence remains limited—retrospective cohorts report inconsistent prognostic associations, and treatment-related changes are poorly characterized. Increased p62 can reflect enhanced synthesis or impaired degradation; mitochondrial clearance can also proceed without it. These observations explain why p62 staining alone cannot measure mitophagic flux. We propose assessing p62 distribution and abundance alongside mitochondrial content, oxidative damage and autophagy–lysosomal markers to describe exploratory tissue profiles. Each profile is linked to a proposed mechanism and to findings that would support or challenge that explanation. We consider baseline response associations separately from changes in residual disease, because complete responders lack residual invasive tumor for paired analysis. Mitochondrial turnover, respiratory function and genetic perturbation with rescue offer complementary ways to test the proposed dependencies. This framework provides a basis for investigating the relationship between mitochondrial damage, clearance, recovery and treatment persistence; its clinical relevance remains to be established.

## Simple Summary

Sequestosome 1 (SQSTM1/p62) is involved in cellular waste handling and stress signaling. It is also measurable in breast cancer tissue, but it is not specific for mitochondrial removal. Human triple-negative breast cancer studies report inconsistent associations between p62 staining and outcome. This narrative review examines what those studies establish and proposes a small set of tissue measurements for future testing. The proposed combinations describe observable patterns of damage, mitochondrial content and stress signaling, but they do not represent validated biological subtypes. Studies should distinguish response prediction based on initial biopsy from adaptation in residual tumors after treatment. Experiments in representative models are then needed to determine whether a tissue pattern corresponds to a mitochondrial dependency.

## 1. Introduction

Triple-negative breast cancer (TNBC) brings together tumors with markedly different morphology, molecular features and treatment responses [1,2]. Estrogen receptor (ER) and progesterone receptor (PR) are each considered negative when fewer than 1% of tumor nuclei stain for the corresponding receptor [3]. Human epidermal growth factor receptor 2 (HER2) negativity is established using the American Society of Clinical Oncology/College of American Pathologists (ASCO/CAP) testing algorithm [4]. ER-low-positive disease should be recorded separately. HER2-low and HER2-ultralow designate levels of HER2 expression and should not replace the receptor criteria used to define TNBC [5].

Understanding how tumor cells manage mitochondrial injury is relevant to the study of their survival during treatment. Here, mitochondrial stress refers to disturbances of mitochondrial homeostasis, including loss of membrane potential, increased mitochondrial oxidant burden, impaired respiration, damage to deoxyribonucleic acid (DNA) or proteins within mitochondria, and changes in organelle architecture. These alterations need not occur together, and oxidative damage staining in tissue does not by itself identify mitochondrial injury. Cells maintain and restore mitochondrial function through protein quality control, biogenesis, fission and fusion, and organelle disposal. Mitophagy, the selective autophagic degradation of mitochondria, is one component of this broader quality control system [6,7,8].

Recent reviews have examined TNBC metabolism, mitochondrial quality control and mitochondrial targeting in detail [6,7,8,9,10]. The present review focuses on how this biology can be investigated through human tissue observations and appropriate experimental models. Pathological complete response (pCR) and residual cancer burden (RCB) provide established response outcomes for such studies [11,12,13]. Interpreting the biology of residual disease also requires a distinction between treatment persistence and resistance. Throughout this review, persistence describes survival during or after a defined exposure and may be reversible; resistance denotes reduced sensitivity demonstrated by response testing or clinical failure. Residual invasive carcinoma alone does not establish stable cell-intrinsic resistance.

Sequestosome 1 (SQSTM1/p62) illustrates the difficulty of moving from a tissue measurement to a mechanistic interpretation. It is detectable in fixed tissue and links cargo organization to redox and nutrient signaling [14,15,16]. Human evidence in TNBC remains limited, with differences in scoring methods, populations and treatment settings that complicate comparison across studies [17,18,19]. Mitophagy can also proceed without p62 in experimental systems [20,21]. We therefore consider p62 alongside measurements of mitochondrial content and damage, defining candidate tissue profiles and the experimental findings needed to support or refute their proposed mechanisms. The resulting profiles are research hypotheses whose reproducibility, functional basis and clinical relevance require independent evaluation.

### Selective Literature Approach and Evidence Appraisal

This is a selective critical narrative review, not a systematic review. Sources were identified through targeted web searches for PubMed-indexed records, examination of primary publications and citation tracing, covering literature available through 1 November 2026. The searches emphasized p62 immunohistochemistry in human TNBC, mitochondrial quality control mechanisms and tissue assay methodology. Part A of Supplementary Table S1 records the search formulations and selection boundaries. These formulations document the discovery route and are not presented as an exhaustive PubMed database search. English-language reports were used; systematic translation and language-independent retrieval were not undertaken.

Original human TNBC studies were prioritized for tissue claims and original perturbation studies for mechanistic interpretation. Mixed breast cancer cohorts, non-TNBC experiments and methodological studies were included when they addressed a specific question relevant to the review, with their limited roles stated explicitly. Reviews provided background and helped identify primary sources but were not treated as independent validation of their findings. Reports unrelated to p62, mitochondrial quality control, TNBC pathology or a defined measurement problem were outside the scope. Selection was non-exhaustive. Complete logs of retrieved records, duplicate handling and study exclusions were not maintained, and dual independent screening was not performed. The bibliography therefore records cited publications rather than a retrieval or inclusion denominator.

Evidence was appraised qualitatively according to population and specimen directness, treatment context, analytical reproducibility, dynamic measurements, causal perturbation and rescue, statistical support, and independent validation (Supplementary Table S1, Part B). These dimensions guided source-specific interpretation; no complete matrix of categorical ratings or numerical appraisal score was generated. A human association may be directly relevant to disease yet provide limited mechanistic insight; a genetic rescue experiment may provide strong mechanistic evidence in a model while remaining clinically indirect. No patient-level reanalysis or meta-analysis was performed.

## 2. p62 Within the Mitochondrial Quality Control Network

### 2.1. p62 Functions Relevant to Tissue Interpretation

**Figure 1:**
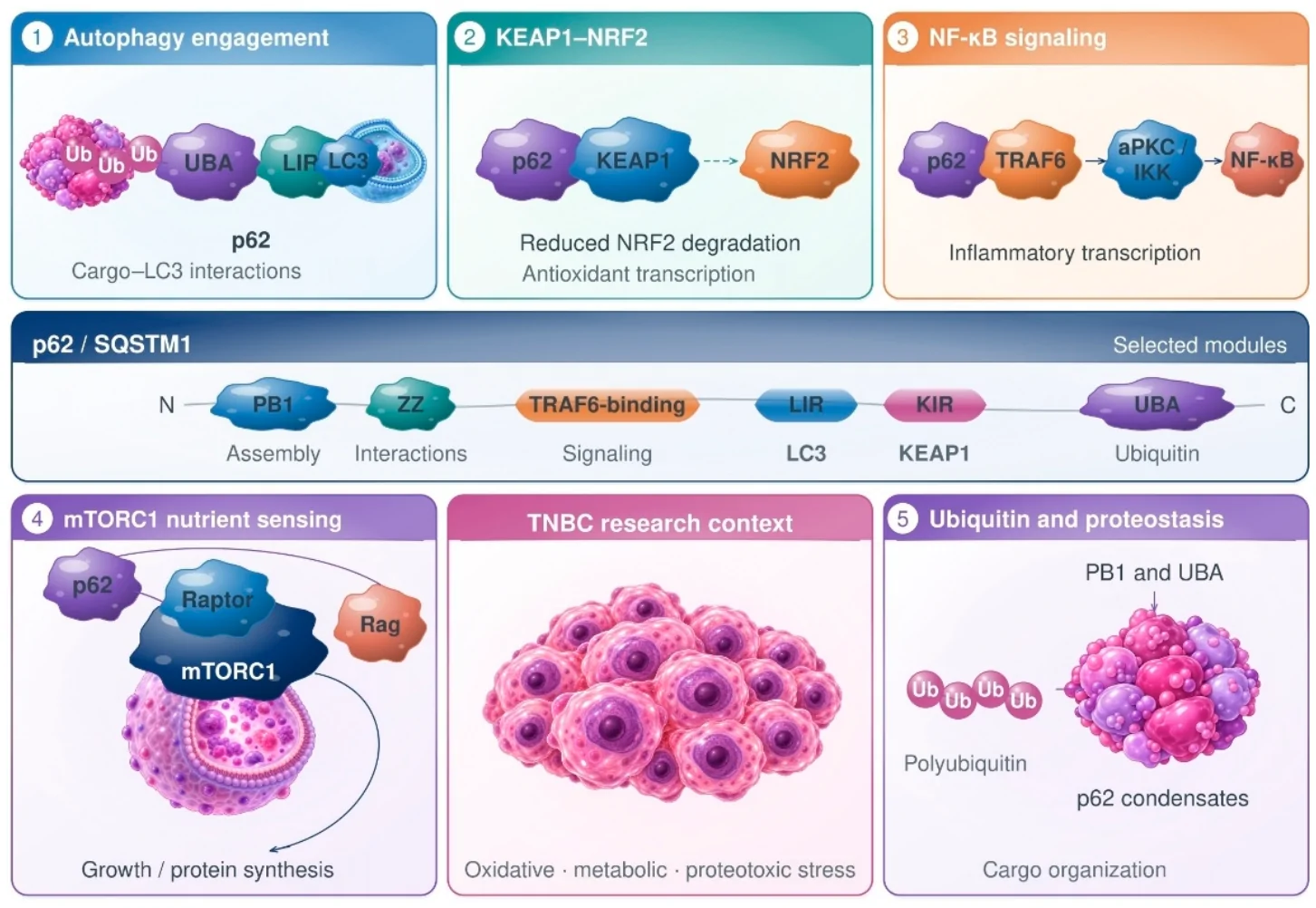
Selected p62 interactions and signaling pathways. Blue marks autophagy engagement, teal marks KEAP1–NRF2 signaling and orange marks NF-κB signaling. Purple identifies the nutrient-sensing and proteostasis panels, and magenta identifies the TNBC context. The strip arranges domains and binding regions in sequence order without representing their relative lengths or residue coordinates [14,15,16,23,24]. The numbered scenes illustrate (1) engagement of ubiquitin-linked cargo with p62 and LC3 [23,24]; (2) KIR–KEAP1 binding and indirect stabilization of NRF2 [22]; (3) TRAF6-associated aPKC/IKK signaling to NF-κB [26]; (4) separate interactions of p62 with Raptor and Rag in amino-acid-dependent mTORC1 signaling at the lysosomal surface [25]; and (5) PB1/UBA-associated cargo condensation and processing [23,24,27]. Rag regulates mTORC1 recruitment but is not a core subunit of the complex. The TNBC illustration provides the disease context and does not indicate tumor-specific validation of these interactions. Clustering mediated by p62 does not establish that p62 is required for mitochondrial degradation [20]. Alternative receptor mechanisms include OPTN/NDP52, BNIP3/NIX and FUNDC1 [21,29,30,31]. Supplementary Table S2 identifies the sources and evidence limits for individual connectors. Abbreviations: p62, sequestosome 1 protein; *SQSTM1*, the gene encoding p62; Ub, ubiquitin; PB1, Phox and Bem1 protein-interaction domain; ZZ, ZZ-type zinc-finger domain (conventional domain designation); UBA, ubiquitin-associated domain; LIR, LC3-interacting region; KIR, KEAP1-interacting region; LC3, microtubule-associated protein 1 light chain 3; KEAP1, Kelch-like ECH-associated protein 1; NRF2, nuclear factor erythroid 2-related factor 2; TRAF6, tumor necrosis factor receptor-associated factor 6; aPKC, atypical protein kinase C; IKK, IκB kinase (IκB, inhibitor of nuclear factor κB); NF-κB, nuclear factor kappa-light-chain-enhancer of activated B cells; mTORC1, mechanistic target of rapamycin complex 1; Raptor, regulatory-associated protein of mechanistic target of rapamycin complex 1; Rag, Ras-related guanosine triphosphate-binding proteins; TNBC, triple-negative breast cancer; OPTN, optineurin; NDP52, nuclear dot protein 52; BNIP3, B-cell lymphoma 2-interacting protein 3; NIX, B-cell lymphoma 2-interacting protein 3-like protein; FUNDC1, FUN14 domain-containing protein 1. N and C indicate the amino and carboxyl termini, respectively.

p62 uses distinct structural regions to organize cargo and engage its binding partners. Its Phox and Bem1 (PB1) domain mediates oligomerization, and the ubiquitin-associated domain binds ubiquitinated cargo. Binding to microtubule-associated protein 1 light chain 3 (LC3) and gamma-aminobutyric acid type A receptor-associated protein (GABARAP) family proteins is mediated by the LC3-interacting region (LIR). The region that binds Kelch-like ECH-associated protein 1 (KEAP1) connects cargo organization with nuclear factor erythroid 2-related factor 2 (NRF2) signaling [22,23,24]. Other interactions link p62 to nutrient sensing by mechanistic target of rapamycin complex 1 (mTORC1) [25]. In experimental systems, p62 also couples tumor necrosis factor receptor-associated factor 6 (TRAF6) signaling to the activation of the nuclear factor kappa-light-chain-enhancer of activated B cells (NF-κB) through atypical protein kinase C (aPKC) and the IκB (inhibitor of NF-κB) kinase (IKK) complex [26]. Polyubiquitin promotes p62 condensation, while phosphorylation alters cargo binding and condensate processing [23,24,27]. Figure 1 summarizes this experimental evidence, which should not be read as a TNBC-specific validation of every interaction.

At steady state, p62 abundance and localization reflect its synthesis, degradation, redistribution and organization into condensates. Phosphorylation at serine residue 403 (Ser403) alters the handling of ubiquitinated cargo without identifying that cargo as mitochondrial [22,23,24,27,28]. Similar staining patterns can therefore arise from different biological processes. Pathological assessment should distinguish cellular and subcellular compartments and interpret p62 alongside independent measurements of damage and mitochondrial content. Staining categories alone cannot resolve turnover; the experiments needed to assess it are described in Section 6.

### 2.2. Ubiquitin-Dependent Recruitment and Mitochondrial Receptor Pathways

When mitophagy is triggered after mitochondrial depolarization through phosphatase and tensin homolog (PTEN)-induced kinase 1 (PINK1) and the ubiquitin ligase Parkin, ubiquitin signals recruit receptors such as optineurin (OPTN) and nuclear dot protein 52 (NDP52), also known as calcium-binding and coiled-coil domain-containing protein 2 (CALCOCO2). Knockout and reconstitution experiments identify these proteins as important recruiters of the autophagy machinery [21]. p62 can cluster damaged mitochondria without being required for their degradation [20], a distinction between organization and disposal that is also addressed by recent condensation studies [32]. Mechanistic validation should therefore include OPTN and NDP52 even when practical considerations favor p62 as an exploratory tissue stain.

B-cell lymphoma 2-interacting protein 3 (BNIP3) and B-cell lymphoma 2-interacting protein 3-like protein (BNIP3L, also known as NIX) engage the autophagy machinery at mitochondria without requiring the same Parkin-dependent ubiquitin cascade. NIX receptor function was established in experimental systems outside TNBC [30]. In a mammary tumor model, *Bnip3* loss increased abnormal mitochondrial accumulation and tumor progression, with re-expression providing support for a causal relationship [29]. The human analyses in that study examined copy number and expression, including 113 TNBC cases in the copy number comparison and a 101-case TNBC expression–survival series. These observations do not validate BNIP3 immunohistochemistry (IHC) in human TNBC. FUN14 domain-containing protein 1 (FUNDC1) mediates another hypoxia-regulated receptor route described in non-TNBC experimental systems [31]. These alternative pathways explain why a negative PINK1/Parkin or p62 result does not exclude mitochondrial disposal.

Mitochondrial clearance also includes routes that export material from cells without lysosomal destruction. Vykoukal et al. demonstrated vesicle-associated clearance in TNBC models and linked its disruption to changes in sphingolipids, respiration and tumor growth [33]. Distinguishing intracellular lysosomal delivery from extracellular export and transfer to neighboring cells is therefore essential when assigning a clearance mechanism.

## 3. Human Evidence in TNBC

### 3.1. p62 Immunohistochemistry in Human TNBC

Luo et al. assessed p62 in 163 TNBCs using tissue microarrays with triplicate cores from each tumor and classified expression as high in 51 cases (31.3%). Ten-year disease-free survival was 54.6% in the high-expression group and 78.2% in the low-expression group; the corresponding distant metastasis-free survival estimates were 58.9% and 92.5% [17]. These findings associate p62 staining with outcome in a retrospective cohort, but mitochondrial turnover was not measured. The study used clone D-3 and an intensity–proportion score, although the Methods and Results describe cutoff selection differently. Assessment by a single reader, tissue-microarray sampling and historical receptor definitions limit the application of these categories to other cohorts.

Kim et al. examined 119 TNBCs and found higher nuclear p62 in the molecular-apocrine and claudin-low categories. Their multivariable analysis associated p62 negativity with shorter disease-free and overall survival [18], a direction that differs from the findings of Luo et al. The studies assessed different compartments and used different scoring systems and case definitions, so they do not support a common high-versus-low threshold. A mixed series of 489 breast cancers also showed that autophagy marker expression varied by subtype and compartment [34]. That study provides context for interpreting staining patterns, rather than an independent TNBC-specific validation of mitophagy.

Wang et al. examined 107 breast cancers, including 31 TNBCs, before neoadjuvant chemotherapy and in the residual tumors available after treatment [19]. In total, 12 TNBC patients achieved study-defined pCR (Grade 3), while 19 were classified as non-pCR. Baseline p62 categories were available for 30 TNBCs (20 high and 10 low), compared with 19 after treatment (9 high and 10 low). Because these denominators differ, the category counts cannot be interpreted as a within-patient decrease in p62. The report provided neither a TNBC-specific paired effect size nor a paired-change test. Its recurrence model combined breast cancer subtypes, and the adjusted association with p62 did not reach statistical significance. The study therefore supports the feasibility of examining p62 before and after treatment, while leaving the direction and magnitude of TNBC-specific adaptation unresolved.

### 3.2. Complementary Human Studies and Limits of Interpretation

Lee H. et al. assessed nuclear and cytoplasmic p62 in 439 TNBC resections from patients who had not received neoadjuvant therapy. The material comprised temporal cohorts of 218 and 221 cases [35], and cytoplasmic p62 correlated with cyclooxygenase-2 (COX-2) in both. These are static associations between markers of cellular stress and autophagy. The same publication also examined 37 patient-derived xenograft (PDX) tumors; those findings come from models and should be considered separately from the clinical resection findings.

Other human studies provide information about redox responses. Nuclear NRF2 and cytoplasmic KEAP1 were examined in operable TNBC [36], while another series related pretreatment phosphorylated NRF2 to response after neoadjuvant therapy [37]. These observations do not by themselves show that p62 activates NRF2 or that mitochondria are the source of the oxidants. A further study assessed hypoxia- and autophagy-related stains in pretreatment biopsies from 57 TNBC patients receiving dose-dense doxorubicin/cyclophosphamide plus paclitaxel, with or without carboplatin [38]. The exploratory response associations in the pooled cohort were not significant within the individual regimen strata and do not establish mitophagy. Complementary evidence comes from the human genomic and transcriptomic *BNIP3* analyses reported by Chourasia et al. [29].

Taddei et al. examined nuclear NRF2 staining and MET receptor tyrosine kinase expression by IHC in 15 TNBC biopsies [39]. In a separate publication, a reanalysis of the PROMIX dataset of HER2-negative breast cancers found oxidative phosphorylation (OXPHOS) enrichment in a subset of residual tumors with suppression of *MYC*, a proto-oncogene encoding a transcription factor [40]. The same publication reported similar B-cell lymphoma-extra-large protein (BCL-XL) staining in paired baseline and residual tumors from 10 patients treated with anthracycline-based neoadjuvant therapy.

In 79 TNBCs treated with doxorubicin/cyclophosphamide followed by paclitaxel, Liu et al. associated higher tumor necrosis factor receptor superfamily member 19 (TNFRSF19) IHC with pCR and Miller–Payne grades 4–5 [41]. The study defined pCR as ypT0 ypN0: no residual invasive or in situ breast carcinoma, and no regional nodal metastasis after neoadjuvant treatment. Promoter methylation was measured by quantitative methylation-specific polymerase chain reaction (qMSP) in the same institutional cohort. Survival associations in public gene-expression datasets were analyzed separately. These associations do not establish differential treatment benefit.

Further studies associated *SQSTM1* expression with epithelial–mesenchymal transition (EMT)/stemness [42] and developed a mitophagy-related prognostic signature [43]. These expression-based findings address different questions from protein localization or mitochondrial turnover. Their independence as evidence depends on checking for overlap between the public datasets. Supplementary Table S3 summarizes the selected evidence by type. Table 1 presents the findings from human tissue and molecular studies.

## 4. Exploratory Tissue Measurements and Candidate Profiles

### 4.1. Morphology and Compartment-Specific p62 Assessment

Morphological assessment begins with hematoxylin and eosin (H&E) examination to identify viable invasive carcinoma, necrosis, stroma, inflammatory cells and treatment effects. High grade, geographic necrosis and a lymphocyte-rich border can be seen in basal-like and triple-negative breast cancers [46,47], but these features do not define a mitochondrial state. Apocrine differentiation also warrants attention because granular eosinophilic cytoplasm may already be a feature of the tumor at baseline; its distinctive clinicopathological context is illustrated by a series of 42 cases [48]. Histological type and diagnostic mimics also guide the choice of further immunohistochemical and molecular tests [49].

Cytoplasmic eosinophilia is a morphological observation; attributing it to mitochondrial enrichment requires organelle-specific evidence [50]. Three-dimensional (3D) electron microscopy has also documented mitochondrial remodeling in TNBC PDXs [51], although these remain model observations rather than serial measurements in patients.

**Figure 2:**
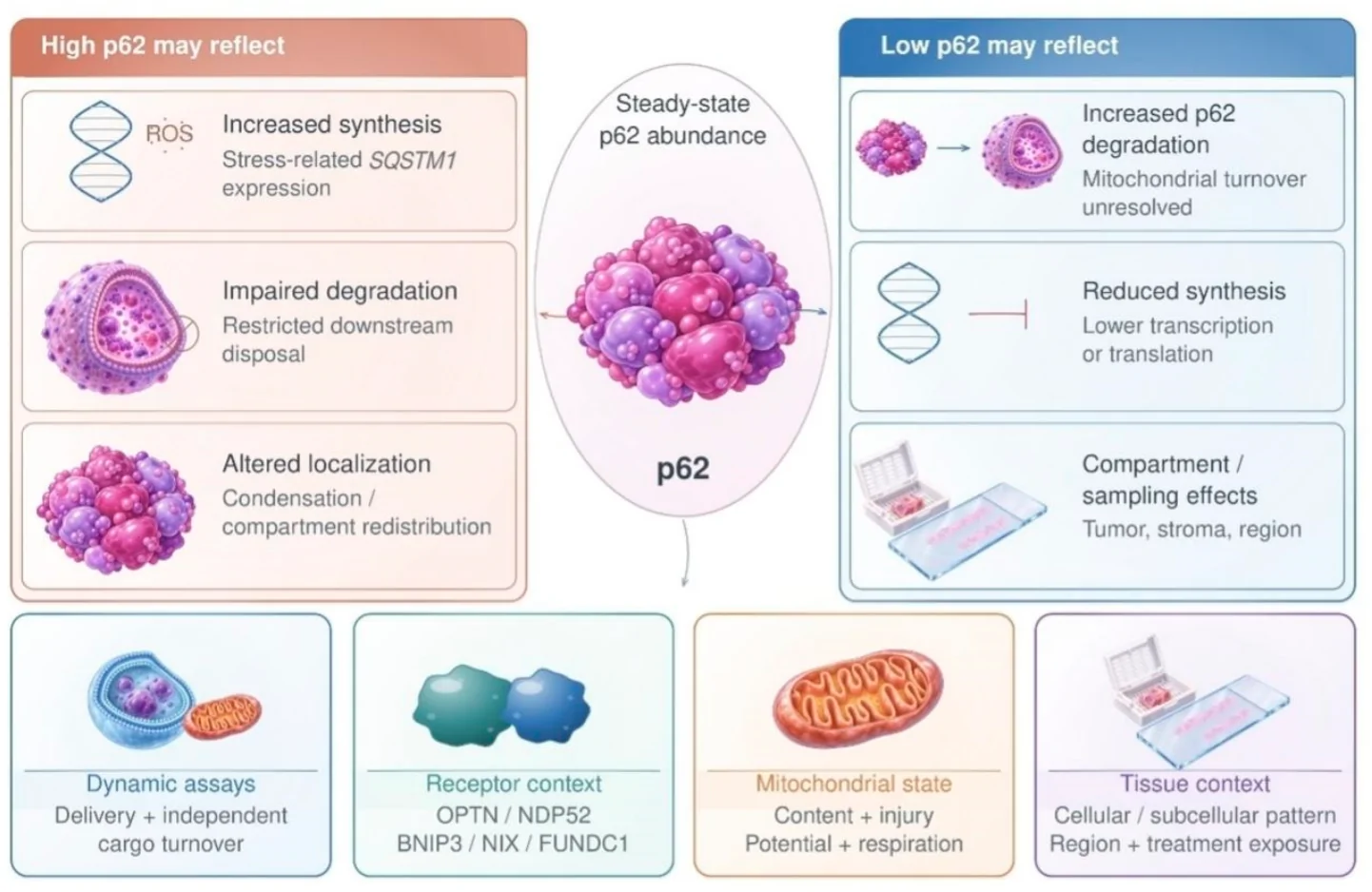
Competing explanations for steady-state p62 abundance. Orange and blue identify possible explanations for high and low p62, respectively. High p62 can reflect increased synthesis, impaired degradation or changes in localization and condensation; low p62 can reflect increased p62 degradation, reduced synthesis or compartment and sampling effects [22,23,24,27,28,55]. Neither high nor low p62 alone establishes mitochondrial turnover or a treatment dependency. The lower panels outline the complementary evidence needed to distinguish these explanations: dynamic measurements of mitochondrial delivery and cargo turnover in living models, pathway-specific receptor context, mitochondrial content and function, and tumor cell, spatial and treatment context [20,21,28,29,30,31,56,57,58]. Arrows organize the competing explanations and the tests used to investigate them; they do not assign a mechanism to an individual tumor. Abbreviations: p62, sequestosome 1 protein; *SQSTM1*, the gene encoding p62; ROS, reactive oxygen species; OPTN, optineurin; NDP52, nuclear dot protein 52; BNIP3, B-cell lymphoma 2-interacting protein 3; NIX, B-cell lymphoma 2-interacting protein 3-like protein; FUNDC1, FUN14 domain-containing protein 1.

A useful p62 assessment distinguishes staining in tumor epithelium, stroma and immune cells. Within tumor cells, nuclear staining, diffuse cytoplasmic staining, puncta and larger inclusions represent separate patterns. Possible exploratory measurements include a continuous cytoplasmic H-score (0–300), the percentage of nuclear-positive cells and the density of puncta per viable tumor area. Defining the cell population, numerator and denominator makes these measurements interpretable and distinguishes cell-based proportions from area-normalized measurements [52]. Nuclear positivity and puncta density therefore remain separate variables. These p62 scoring systems have not been clinically validated. Nuclear and cytoplasmic p62 patterns have been recorded in meningioma and chordoma [53,54], but their cutoffs and prognostic associations cannot be transferred to TNBC. Figure 2 brings together the possible explanations for high and low p62 measurements and the independent evidence needed to distinguish them.

A reproducibility study would use at least two blinded observers to score prespecified regions after calibration with reference images. Absolute-agreement intraclass correlation coefficients (ICCs), reported with confidence intervals, quantify agreement for continuous measurements; weighted kappa applies to ordered categories. Repeat scoring and changes in region selection help identify sources of variability. Digital segmentation requires an independent tumor mask and manual review of errors, particularly in necrotic or lymphocyte-rich areas. The proportion of interpretable measurements is reported separately from agreement among evaluable specimens. These performance estimates require testing directly in TNBC material.

In metastatic specimens, mammary origin should be confirmed through clinicopathological assessment before stress markers are interpreted. Combined staining for transcriptional repressor GATA binding 1 (TRPS1) and GATA-binding protein 3 (GATA3) can support this lineage assessment in ER-negative or ER-low breast carcinoma [59].

### 4.2. Candidate Panel and Analytical Qualification

An initial exploratory panel could combine compartment-specific p62 with microtubule-associated protein 1 light chain 3 beta (LC3B) assessment. Mitochondrial content could be assessed using translocase of outer mitochondrial membrane 20 (TOMM20/TOM20), with cytochrome c oxidase subunit IV (COX IV) as an orthogonal marker. An oxidative-damage measure such as 4-hydroxy-2-nonenal (4-HNE) or 8-hydroxy-2′-deoxyguanosine (8-OHdG) could be added alongside nuclear NRF2. Lysosome-associated membrane protein 1 (LAMP1) would provide the corresponding compartment context. Additional measurements could include phospho-p62 Ser403 and BNIP3/NIX once antibody performance has been established, with verification of phosphoepitope preservation for phospho-p62. The inclusion of OPTN, NDP52, FUNDC1, PINK1, Parkin, dynamin-related protein 1 (DRP1) or optic atrophy 1 mitochondrial dynamin-like protein (OPA1) should depend on the mechanism under investigation; these markers need not be applied to every biopsy.

The LC3 isoform and staining pattern determine what is being scored. The “stone-like” structures associated with the alpha isoform of LC3 (LC3A) in endometrial pathology [60,61] differ from the LC3B puncta proposed here; combining them would obscure that distinction.

Commercial availability or use in a previous publication is insufficient to establish an antibody’s analytical validity in formalin-fixed, paraffin-embedded (FFPE) TNBC. Qualification examines localization, agreement with immunoblotting or another independent assay, positive and negative controls, variation between lots and platforms, and sensitivity to ischemia and fixation. Genetically depleted and reconstituted FFPE cell blocks provide useful controls where feasible, with phosphatase-sensitive controls for phosphorylation-specific staining. The comparison of TOMM20 and COX IV with independent measures of mitochondrial content is particularly important: altered protein expression or selective membrane protein degradation can change either stain without a proportional loss of organelles. Neither stain establishes respiratory competence.

Documented cold ischemia, fixation and block storage conditions help assess pre-analytical variation. Staining paired core-biopsy and resection specimens in balanced batches with shared controls addresses an additional source of variability. Interpretation also depends on what each assay measures. Total DRP1 staining does not measure fission activity, while total OPA1 staining does not resolve protein processing or establish fusion competence. PINK1 stabilization and Parkin recruitment to mitochondria are difficult to establish from diffuse FFPE immunoreactivity. NRF2 is interpreted through its nuclear staining, whereas KEAP1 abundance alone cannot demonstrate functional sequestration by p62. No antibody clone or diagnostic cutoff is endorsed for the exploratory panel; published reagents are provided as source identifiers to support reproducibility. Table 2 summarizes the exploratory status of each candidate measurement.

### 4.3. Minimum Profile Definitions and Falsification Criteria

A candidate tissue profile combines measurements of mitochondrial content, damage and compartment-specific stress or autophagy in the same registered region of viable tumor. Profiles A and C require concordant TOMM20 and COX IV results. Profile B uses a qualified TOMM20 or COX IV assay selected before analysis; when both are measured, content is indeterminate if one is reduced and the other is preserved or increased. Increased 4-HNE or 8-OHdG provides the oxidative damage criterion. The attribution of that damage to mitochondria requires validated subcellular colocalization; the registration of serial sections establishes correspondence between regions, not between individual organelles. The profile names describe the observed tissue pattern, while functional experiments test its proposed mechanism.

**Figure 3:**
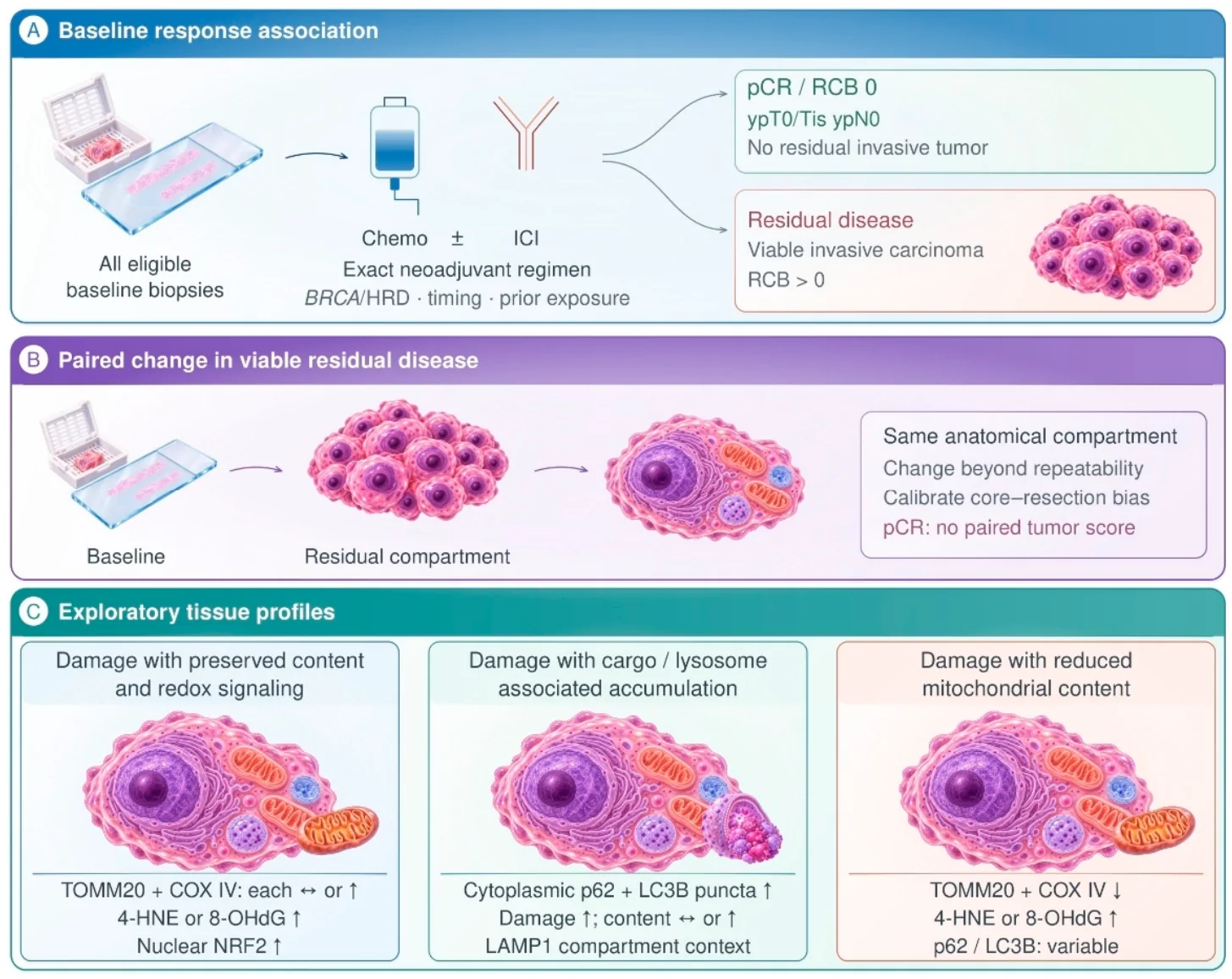
Separate study designs for baseline response and residual disease. (**A**) Pretreatment features are examined for associations with pCR and RCB under a defined neoadjuvant regimen. Here, pCR is ypT0/Tis ypN0, corresponding to RCB 0 [11,12,13,65]. (**B**) Paired analysis requires adequate viable carcinoma from the same anatomical compartment at both time points. Residual disease confined to lymph nodes does not provide a paired-change score for the breast. Observed differences can reflect adaptation, selection or sampling; bias from comparing core biopsies with resections requires independent calibration. (**C**) Three exploratory profiles, which may overlap, specify expected marker directions. Table 3 defines marker requirements, proposed dependencies and falsification criteria. Marker directions are assessed in registered viable-tumor regions against a predefined reference or matched baseline, with normalization to viable cells. Mitochondrial attribution of damage requires colocalization. No validated cutoffs are available. Active delivery and impaired disposal remain alternative explanations for the middle profile. Arrows in (**A**,**B**) indicate the study sequence. In (**C**), ↑, ↓ and ↔ indicate increased, decreased and preserved marker levels, respectively. The blue, purple and teal headers identify panels (**A**–**C**), respectively. Abbreviations: Chemo, chemotherapy; ICI, immune checkpoint inhibitor; *BRCA*, breast cancer susceptibility genes 1 and 2; HRD, homologous recombination deficiency; pCR, pathological complete response; RCB, residual cancer burden; TOMM20, translocase of outer mitochondrial membrane 20; COX IV, cytochrome c oxidase subunit IV; 4-HNE, 4-hydroxy-2-nonenal; 8-OHdG, 8-hydroxy-2′-deoxyguanosine; NRF2, nuclear factor erythroid 2-related factor 2; p62, sequestosome 1 protein; LC3B, microtubule-associated protein 1 light chain 3 beta; LAMP1, lysosome-associated membrane protein 1. ypT0/Tis ypN0 denotes post-neoadjuvant pathological stage with no residual invasive breast carcinoma (in situ disease may remain) and no regional nodal metastasis.

The three research profiles in Table 3 describe damage accompanied by preserved or enriched mitochondrial content and redox signaling, damage accompanied by cargo or lysosome-associated accumulation, and damage accompanied by reduced mitochondrial content. Their expected marker directions refer to a predefined reference distribution or a matched baseline. Continuous measurements are preferable during assay development. Where research categories are needed, their reference percentiles are fixed in a training set before outcome testing; a paired change qualifies only if it exceeds the assay-specific 95% repeatability limit established from replicate measurements. Any resulting thresholds require independent validation, and no universal numerical cutoff for p62 or mitochondrial stress is currently supported. Figure 3 shows how the profiles can be investigated in separate baseline and residual-disease study designs.

A tumor can contain more than one profile, so classification applies to registered regions of viable carcinoma.

## 5. Regimen-Specific Experimental Evidence

### 5.1. Chemotherapy and Mitochondrial Adaptation

Studies in cell lines and patient-derived models show distinct responses to cytotoxic treatment. Baek et al. linked survival after DNA-damaging chemotherapy to mitochondrial elongation associated with OPA1, increased oxidative metabolism and regrowth [63]. After anthracycline exposure, Andrzejewski et al. identified an OXPHOS-high state dependent on BCL-XL. Responses varied across cell lines and differed from those in cells persisting after taxanes [40]. Respiratory vulnerability has been demonstrated in selected TNBC models [45,66], but greater mitochondrial content alone does not establish respiratory dependence.

The PDX study using 3D electron microscopy reconstructed 3750 mitochondria and 800 lipid droplets across models and treatment conditions [51]. These are nested organelle observations, not independent patients or animals. Another PDX study linked a basal population enriched in chromosome 15 open reading frame 48 (*C15ORF48*) to mitochondrial reactive oxygen species (ROS) regulation after doxorubicin/cyclophosphamide [67]. Studies of giant-cell states induced by paclitaxel [68], cells tolerant to doxorubicin or paclitaxel [64], and MDA-MB-231 cells depleted of transient receptor potential mucolipin 1 (TRPML1) [69] addressed different exposures and selection histories. Their autophagy and lysosome findings should not be pooled as a single validated TNBC phenotype.

In breast cancer models, Parkin suppression or microRNA-218-5p (miR-218-5p) enhanced the effects of doxorubicin [70]. Experiments involving TNFRSF19 suggest a separate mitophagy hypothesis specific to doxorubicin [41]. Polyphyllin I induced mitochondrial injury and changes associated with mitophagy, yet PINK1 depletion increased apoptosis [71], consistent with a protective contribution from clearance. Isorhamnetin combined with chloroquine linked mitochondrial fission and apoptosis through signaling involving ROS, calcium/calmodulin-dependent protein kinase II (CaMKII) and DRP1 [72]. This association does not establish excessive mitochondrial clearance as the cause of death.

### 5.2. Non-Mitochondrial p62 Functions and Resistance to Poly(adenosine Diphosphate-Ribose) Polymerase Inhibitors

Post-transcriptional regulation can raise p62 without impaired degradation. An *SQSTM1* transcript with a short 3′ untranslated region (UTR) promoted chemoresistance in breast cancer [55]. Autophagy inhibition can also generate condensates containing the NBR1 autophagy cargo receptor and p62, affecting itchy E3 ubiquitin-protein ligase (ITCH), tumor protein p63 and metastatic programs [73].

In cells lacking secretory carrier membrane protein 3 (SCAMP3), the inhibition of extracellular signal-regulated kinases 1 and 2 (ERK1/2) increased p62 and lipidated LC3B [74]. The peptide WK-13-3D impaired bulk autophagic flux and p62 degradation [75], while experiments with compound 7h associated nuclear p62 accumulation with impaired DNA repair [76]. These studies show how changes in p62 can arise from processes outside mitochondrial quality control.

Steady-state p62 was reduced in olaparib-resistant SUM159 and MDA-MB-468 models. Both had intact breast cancer susceptibility genes 1 and 2 (*BRCA1/2*), although homologous recombination deficiency (HRD) was reported for MDA-MB-468 [77]. Nitro-oleic acid experiments included resistant TNBC models with mutant *BRCA1* and implicated p62 modification at cysteine residues 105 and 113 (Cys105/Cys113) and disruption of the autophagy–lysosome system [78]. These studies address different molecular contexts. Neither establishes a tissue p62 cutoff that predicts benefit from poly(adenosine diphosphate-ribose) polymerase (PARP) inhibition in patients.

### 5.3. Immunotherapy and Radiotherapy: Distinct Treatment Contexts

In TNBC models, the p62-dependent degradation of tenascin-C supports T-cell-mediated killing [44]. The cargo in this mechanism is an extracellular matrix protein, so the finding does not identify a mitophagic mechanism of checkpoint-inhibitor response. Human spatial analyses such as NeoTRIP examine response to specific neoadjuvant chemotherapy and immunotherapy combinations [79]. Evidence on survival with immunochemotherapy in advanced or metastatic disease addresses a different clinical question [80]. Programmed death-ligand 1 (PD-L1) assessment in metastatic TNBC also depends on the assay and the cell compartments included in scoring [81].

Radiotherapy studies implicate the HECT-domain E3 ubiquitin-protein ligase 3 (HECTD3; HECT, homologous to the E6-associated protein carboxyl terminus)–p62 axis and a program involving p62 and vascular cell adhesion molecule 1 (VCAM-1) [82,83]. These model studies connect p62-associated signaling with DNA repair and general autophagy during radiation responses. Comparisons between biomarker studies also depend on clearly defined treatment and sampling times [84].

### 5.4. Therapeutic Hypotheses and the Standard for Lethal Clearance

Studies in experimental models have tested respiratory inhibition, lysosomal disruption, signaling involving MET, the non-receptor tyrosine kinase SRC and NRF2, and drug delivery targeting mitochondria as treatment strategies [39,45,85,86].

In a study of carboplatin, the depletion of autophagy-related protein 5 (ATG5) did not change sensitivity to carboplatin or BMS-754807 [85]. Autophagy inhibition alone therefore did not explain the effects of hydroxychloroquine.

Bi-magnolignan studies combined measurements of mitochondrial delivery using the mitochondria-targeted Keima (mt-Keima) reporter with ultrastructural analysis, the assessment of depolarization and apoptosis, and the perturbation of TANK-binding kinase 1 (TBK1; TANK, tumor necrosis factor receptor-associated factor family member-associated NF-κB activator) [62]. Studies of redox-responsive conjugates also linked mitochondrial injury and mitophagy-associated measurements to antitumor effects [87]. Whether clearance contributes to cell death remains a hypothesis, because TBK1 pleiotropy and other drug effects offer alternative explanations. A causal interpretation requires selective clearance blockade to preserve mitochondrial mass, respiration and long-term viability under comparable initial exposure and injury. Restoring the targeted pathway should then reinstate mitochondrial clearance and cell death (Section 6). Table 4 compares selected experiments by model, exposure, endpoint and strength of causal evidence.

## 6. Functional Testing of Tissue-Based Hypotheses

### 6.1. Measuring Mitochondrial Delivery and Turnover

**Figure 4:**
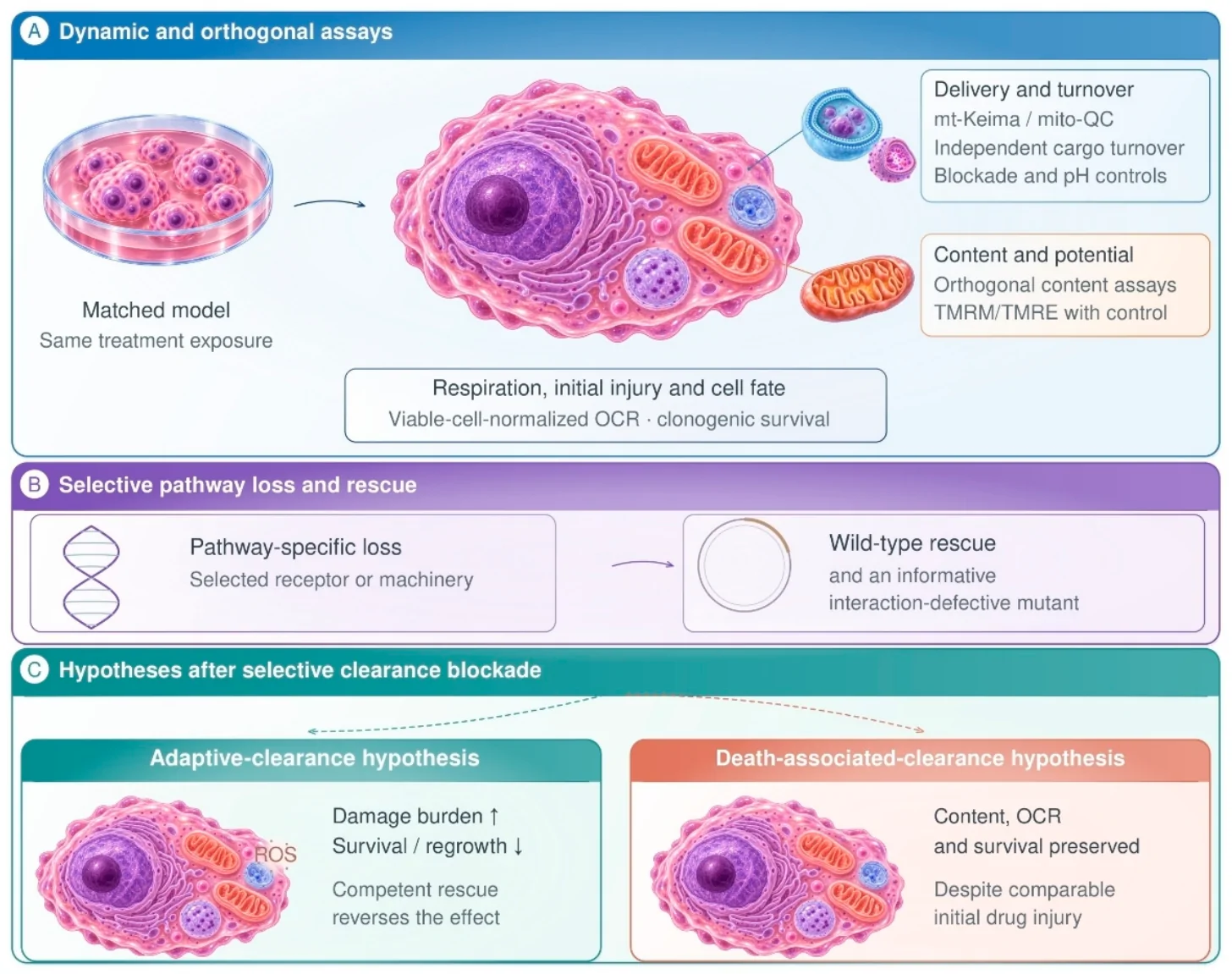
Functional testing of a candidate tissue profile. (**A**) Models matched to the tumor and treatment context combine time-resolved measurements of mitochondrial delivery and cargo turnover with independent assays of content, membrane potential, respiration, injury and survival [28,56,57,58]. Reporter interpretation requires pH and proteolytic controls and independent cargo-turnover measurements, because lysosomal alkalinization changes reporter signals. (**B**) Pathway-specific loss-of-function experiments, rescue with a functional wild-type construct and informative mutants test the proposed dependency [20,21,29]. (**C**) Dashed arrows lead to the adaptive-clearance hypothesis (teal) and the death-associated-clearance hypothesis (orange). Upward and downward arrows denote increases and decreases, respectively. Selective clearance blockade is expected to impair survival when clearance is adaptive, or to preserve mitochondrial content, respiration and survival when clearance contributes to death. These comparisons require comparable initial drug injury; a discordant response challenges the proposed interpretation. Abbreviations: mt-Keima, mitochondria-targeted Keima fluorescent reporter; mito-QC, mitochondrial quality control fluorescent reporter; pH, measure of acidity defined by the negative base-10 logarithm of hydrogen-ion activity; TMRM, tetramethylrhodamine methyl ester; TMRE, tetramethylrhodamine ethyl ester; OCR, oxygen-consumption rate; ROS, reactive oxygen species; TNBC, triple-negative breast cancer.

Functional validation asks whether a tissue pattern corresponds to its proposed mechanism (Figure 4), using models matched to the source tumor compartment, molecular context and exact treatment exposure. Time-resolved mt-Keima or the mitochondrial quality control reporter (mito-QC) can track mitochondrial delivery into acidic compartments, with signals normalized to viable cells and reporter expression [56,57]. Interpretation requires confirmed mitochondrial targeting, lysosomal colocalization and an established positive/negative dynamic range. These reporters capture delivery and accumulated signal, so a single endpoint cannot provide a complete degradation-rate estimate. Sensitivity also depends on the reporter: a direct comparison found mito-QC less sensitive to events dependent on PINK1 and the Parkin ubiquitin ligase, encoded by *PRKN* [58]. A negative result requires an adequate pathway-specific dynamic range for interpretation. Tandem LC3 reporters assess general autophagy and cannot replace a mitochondrial reporter [28].

Short, titrated lysosomal-blockade controls help interpret mitochondrial cargo turnover and LC3 processing, alongside measurements of lysosomal acidity (pH) and cell toxicity. Bafilomycin and chloroquine alter the acidic environment on which mt-Keima and mito-QC depend, so reporter changes after alkalinization cannot be interpreted by simple inhibitor subtraction. Calibrated reporter kinetics should be considered with independent measurements of matrix and membrane cargo loss/turnover, lysosomal protease controls where appropriate, and the genetic interruption of autophagosome delivery [28,56,57]. Electron microscopy adds structural evidence of sequestration without establishing a rate.

### 6.2. Measuring Mitochondrial Content, Membrane Potential and Respiration

The functional assessment of mitochondrial content combines at least two orthogonal measurements, such as TOMM20/COX IV imaging, the mitochondrial DNA (mtDNA)-to-nuclear DNA ratio and citrate synthase activity. Cell size, ploidy and cell death influence these readouts and their interpretation. Membrane-potential measurements with tetramethylrhodamine methyl ester (TMRM) or tetramethylrhodamine ethyl ester (TMRE) are interpreted against a depolarization control and normalized to mitochondrial content; dye fluorescence is not an independent measure of mass. Respiratory assessment includes appropriately normalized basal, adenosine triphosphate (ATP)-linked and maximal oxygen consumption, spare respiratory capacity and non-mitochondrial oxygen consumption. Compartment-appropriate probes and interference controls distinguish ROS signals from nonspecific oxidation or assay effects [19,28,29,63].

A respiratory dependency is supported when orthogonal respiratory perturbations reduce clonogenic survival or regrowth; high oxygen consumption alone is insufficient. For redox dependence, genetic or pharmacological pathway manipulation is paired with suitable rescue and controls for direct effects of antioxidants on the compound. Replication in more than one biologically relevant model helps establish whether the relationship extends beyond a single experimental system. Animals or independent cultures provide the biological replicates; individual cells and organelles are nested measurements.

### 6.3. Genetic Perturbation, Rescue and Tests of the Proposed Mechanism

The choice of perturbation should follow the pathway observed in the model: PINK1/Parkin, OPTN/NDP52, BNIP3/NIX or FUNDC1, with *SQSTM1* targeted only when p62 dependence is being claimed. Testing pathway redundancy helps clarify the basis of the response. Further support comes from using at least two independent targeting reagents and performing rescue with an appropriately expressed wild-type construct resistant to the targeting reagent. Cargo-binding or LIR-defective mutants can help separate signaling and cargo organization from delivery. The inhibition of ATG5, autophagy-related protein 7 (ATG7) or lysosomal function alone cannot establish that an effect is specific to mitochondria [20,21,28,29,30,31].

An adaptive role for clearance is supported when its selective interruption reduces mitochondrial delivery, increases damaged organelle burden and impairs survival under matched exposure. Restoring the functional pathway should recover delivery and turnover and reverse the survival defect. A lethal-clearance mechanism predicts the opposite survival effect—interruption preserves mitochondrial content, respiration and clonogenic survival despite comparable initial injury, while pathway restoration reinstates clearance and cell death. Failure to observe the predicted survival change after verified blockade challenges the proposed dependency under the conditions tested. Death preceding organelle loss specifically challenges lethal clearance. Rescue through downstream or compensatory signaling alone neither establishes nor excludes clearance-specific causality. Together, these experiments test the damage–clearance–recovery hypothesis under defined experimental conditions.

## 7. Clinical Study Design, Statistical Analysis and Feasibility

### 7.1. Baseline Markers and Treatment Response

Baseline response studies encompass consecutive eligible patients with pretreatment TNBC, including those who subsequently achieve pCR. Here, pCR denotes no residual invasive breast carcinoma (in situ carcinoma may remain) and no regional nodal metastasis after neoadjuvant treatment (ypT0/Tis ypN0), with RCB recorded according to an established protocol [11,12,13]. Logistic regression can relate a prespecified continuous marker or small panel to pCR, with stage, regimen and relevant molecular features chosen in advance. A model for RCB accounts for its distribution, including the concentration of observations at zero in complete responders; one option is a two-part model separating pCR from positive RCB. These analyses estimate associations with response under the regimen studied. Evidence that a marker predicts benefit from one treatment over another instead comes from a comparative design with a prespecified marker-by-treatment interaction.

### 7.2. Sampling and Change in Residual Disease

Paired analyses examine how measured profiles differ after treatment among patients with residual disease. They require adequate viable carcinoma from the same anatomical compartment at both time points. A breast analysis therefore compares the pretreatment breast core with residual invasive breast carcinoma. Patients with residual nodal disease but no invasive breast tumor remain eligible for the baseline outcome analysis, but cannot contribute a breast paired-change measurement. Nodal adaptation requires its own pretreatment nodal specimen and a separately specified post-treatment analysis. The absence of residual tumor has no corresponding marker score and cannot be coded as zero expression. Reporting includes the selection of pairs, comparisons between included and excluded non-pCR patients, and tissue adequacy separately from specimen availability. Weighting can address some measured sources of selection, but cannot recover a post-treatment tumor profile where no tumor remains.

Core biopsies and resections sample different volumes and regions and often undergo different fixation conditions. Multiregion baseline sampling, where feasible, together with consistent cell compartments, viable-tumor masks and replicate regions can help characterize this variability. The original tumor bed, residual cellularity and nodal disease should be documented according to neoadjuvant reporting recommendations [65]. Spatial studies show how tissue composition influences interpretation [79,88,89,90,91,92,93], although exact correspondence between regions before and after treatment remains unavailable. Specimen and patient counts should therefore be reported separately. Even with careful sampling, a paired difference alone cannot distinguish treatment-induced adaptation from the selection of a pre-existing cell population.

Mixed-effects models can account for repeated continuous measurements, regions nested within patients and time points, and identifiable batch or technical effects. A fundamental limitation remains when all baseline samples are cores and all post-treatment samples are resections: specimen type is inseparable from time and treatment, and regression adjustment cannot resolve that confounding. An independent core–resection calibration set or matched processing experiment is needed to estimate the technical contribution. Changes would be reported with confidence intervals and the proportion exceeding analytical repeatability limits. Associations between residual-disease profiles and subsequent outcomes would be evaluated from a prespecified post-surgery landmark, accounting for RCB and other justified covariates. Use of a Cox model requires assessment of the proportional-hazards assumption.

### 7.3. Recording Treatment and Molecular Context

Treatment records need to identify the exact agents, doses and schedules, clinical setting, interval from the last dose to sampling, prior exposures and subsequent therapy. Anthracycline/cyclophosphamide, taxane and platinum components are recorded separately, together with any concurrent checkpoint inhibitor. For PARP inhibitors, the relevant context includes neoadjuvant, adjuvant or metastatic use; germline and somatic *BRCA1/2* status; the HRD assay and cutoff; and previous platinum or PARP-inhibitor exposure. An HRD-positive research category is not equivalent to established *BRCA*-based clinical selection. Radiotherapy records also include dose, fractionation and concurrent drugs. Analyses that combine these exposures need explicit interactions or separate strata to preserve the distinctions between their selective pressures.

### 7.4. Statistical Analysis and Sample-Size Planning

This review involved no new statistical testing; all numerical findings are attributed to the original studies. For future investigations, continuous measurements preserve more information than outcome-optimized cutoffs, and a limited set of candidate parameters helps contain overfitting. Prespecifying the primary profile and endpoint separates confirmatory comparisons from exploratory analyses. Secondary panels call for the control of false discovery, with confidence intervals reported alongside effect estimates. Patient-level bootstrap resampling supports internal validation and correction for optimism; a separate temporal or external cohort assesses performance independently. Both discrimination and calibration are relevant to prediction models. A prespecified missing-data plan distinguishes missing covariates, failed staining and exclusions for insufficient tumor. Multiple imputation is appropriate only when its assumptions are defensible and cannot substitute for a residual tumor that is absent.

A pilot study establishes tissue adequacy and staining success, sensitivity to region selection and repeatability between observers. The practical losses can be substantial; in a pretreatment TNBC study, 24 of 31 *BRCA1*/*BRCA2*-tested women met the specimen, panel and exposure eligibility criteria [94]. That immune/stromal study illustrates eligibility attrition in a selected clinical population, rather than validation of mitochondrial profiles. Qualifying a small core panel before adding receptor or phosphoprotein measurements in informative subsets could conserve biopsy material. Multiplex assays need checks for spectral overlap and antibody interference, while serial sections depend on accurate registration. Once analytical feasibility is established, formal sample-size planning considers outcome frequency, candidate parameter count and anticipated performance, rather than a fixed events-per-variable rule [95]. For external validation, a separate sample size calculation aims to estimate calibration and discrimination with adequate precision.

The number of usable pairs can be far smaller than the number of patients enrolled. For illustration, if 200 patients enter a study, 60% achieve pCR and paired material is inadequate in 15% of the remainder, only 200 × 0.40 × 0.85 = 68 pairs would be available. These are assumed planning values, not observed results or a recommended sample size. Further division by regimen or *BRCA* status, together with a limited number of survival events, would restrict model complexity. Multicenter recruitment and a small panel, developed first analytically and then biologically, are likely to be more feasible than deriving a large multiplex classifier from a small single-center series.

### 7.5. Reporting and Independent Validation

Reproducible reporting allows another laboratory to reconstruct the assay and its interpretation. This requires the antibody clone or catalog number, dilution, retrieval, controls, platform and batch, together with cold ischemia, fixation and block age. Cellular and subcellular compartments, scoring and segmentation rules, region selection, blinding, observer agreement and the origin of each cutoff also need explicit definitions. Clinical annotation includes standard receptor status, stage, morphology, tumor-infiltrating lymphocytes (TILs), treatment, *BRCA*/HRD context and pCR/RCB. Independent clinical validation uses the assay, threshold and analysis plan fixed during development. Comparing the panel with its individual markers under a prespecified decision rule and against the same independent endpoint tests whether combining them adds useful information.

## 8. Limitations and Conclusions

The selected human p62 studies are retrospective, use heterogeneous scoring methods and lack simultaneous measurements of mitochondrial flux. Redox, hypoxia and genomic studies add complementary observations, but do not validate the proposed composite profiles. Experimental findings also depend on the model, culture medium, oxygen tension, treatment exposure and selection history. Broad mitochondrial or lysosomal drugs affect normal tissues and multiple cellular pathways, further complicating translation [96,97]. Representative histological images would help readers compare the proposed patterns with actual tissue appearances; their absence is a limitation of this review.

Pathology can identify reproducible spatial patterns that guide mechanistic testing. Whether mitochondrial quality control changes with treatment in human TNBC remains an open question requiring suitable sampling, clearly defined regimens and independent clinical validation. The damage–clearance–recovery model is a testable hypothesis; its value depends on whether experiments support the predicted dependencies and whether those findings explain clinically relevant differences in residual disease. The proposed profiles have no established role in treatment selection.

## Figures and Tables

**Table 1:** Selected human observations relevant to p62 and companion measurements.

Study and Evidence Component	Human Tissue and Sample Size	Setting and Measurement	Reported Endpoint or Finding	Flux, Causality and Validation Limits
Kim et al. [18] TNBC p62 IHC	Yes; 119 TNBCs; TMA	Retrospective; nuclear/cytoplasmic p62 and autophagy panel	Nuclear p62 varied by surrogate subtype; p62 negativity was associated with shorter DFS/OS	No mitochondrial flux measurement or causal experiment in tissue.
Luo et al. [17] TNBC p62 IHC	Yes; 163 TNBCs; triplicate cores per tumor on TMA	Surgery 2000–2008; p62 D-3	High p62 in 51/163; 10-year DFS 54.6% versus 78.2% with low p62	No tissue flux or causal testing; one reader; inconsistent cutoff descriptions.
Choi et al. [34] Mixed breast IHC	Yes; 489 breast cancers	TMA; tumor/stroma and nuclear/cytoplasmic autophagy markers	Subtype- and compartment-dependent expression; does not provide TNBC-specific p62-outcome validation	No mitochondrial flux measurement.
Wang et al. [19] Neoadjuvant p62 IHC	Yes; 107 breast cancers, 31 TNBCs; baseline p62 results for 30 TNBCs and residual results for 19 TNBCs	Anthracycline/taxane-based mixed cohort	TNBC baseline—20 high/10 low; residual—9 high/10 low; 12 TNBCs with study-defined pCR (Grade 3); individual paired p62 change not reported	No tissue flux measurement or causal testing; unequal denominators; recurrence model not specific to TNBC; adjusted p62 association non-significant.
Lee H. et al. [35] TNBC p62/stress IHC	Yes; 439 TNBC resections; temporal cohorts of 218 and 221; TMA	No neoadjuvant therapy; adjuvant AC ± taxane. Nuclear/cytoplasmic p62; ab56416, 1:10,000	Positive cytoplasmic COX-2–p62 correlations in both cohorts (*p* = 0.002/0.003)	Static association; no human tissue flux or causal test. The PDX findings are reported separately.
Lee Y.S. et al. [36] TNBC redox IHC	Yes; 203 TNBC resections; separate 225-case METABRIC RNA analysis	Resection IHC: 189 cases without and 14 after neoadjuvant chemotherapy; predominantly adjuvant-treated. Nuclear NRF2 and cytoplasmic KEAP1	High nuclear NRF2/low KEAP1 associated with worse survival	No p62-specific mechanism or mitochondrial flux demonstrated. RNA association differs in direction from NRF2 IHC; RNA does not independently validate the IHC panel.
Yu et al. [37] Pretreatment phospho-NRF2	Yes; 59 TNBCs; prospective observational cohort	Stage II/III; TAC × 6 or AC × 4 then paclitaxel × 4; baseline nuclear p-NRF2	High p-NRF2 associated with fewer Miller–Payne IV/V responses; endpoint is not standard pCR	No mitophagy flux or causal perturbation; small single-center series.
Taddei et al. [39] TNBC redox IHC	Yes; 15 TNBC biopsy specimens	Nuclear NRF2 and cytoplasmic MET	Strong nuclear NRF2 staining and positive MET staining in 13 specimens; weak nuclear NRF2 staining and negative MET staining in 2	Small descriptive series; no biopsy mitophagy-flux measurement or causal perturbation.
Liu et al. [41] Institutional TNFRSF19 IHC/methylation	Yes; 79 TNBCs; IHC and FFPE promoter methylation in the same cohort	AC every 3 weeks × 4 (doxorubicin 60 mg/m^2^), then paclitaxel	Higher IHC associated with pCR (ypT0 ypN0) and Miller–Payne 4–5; higher methylation associated with lower protein expression/response	Median IHC cutoff; no tissue flux or causal perturbation.
Liu et al. [41] Public expression-survival component	Human tumor datasets; 113 chemotherapy-treated TNBCs from TCGA, including 42 doxorubicin-treated cases; 51 TNBCs from GSE22226	TCGA chemotherapy annotation; doxorubicin/cyclophosphamide-based GSE22226 treatment	Higher *TNFRSF19* expression associated with better overall survival	Maximally selected expression cutoffs; no marker-by-treatment interaction or IHC-panel validation. The 42 cases are a subset of 113.
Mavili et al. [38] Hypoxia/autophagy IHC	Yes; 57 TNBC pretreatment biopsies	Neoadjuvant ddAC plus paclitaxel, with or without carboplatin; HIF1α, Beclin-1, LC3A, Bcl-2, eIF2α	Exploratory pooled Beclin-1/stromal HIF1α response associations; none significant within regimen strata; no association between the tested markers and survival	No p62 or mitochondrial-turnover measurement; small regimen subgroups and internally derived thresholds.
Li et al. [44] Non-mitochondrial immune context	Yes; 160 TNBCs for TNC IHC; LC3B correlation *n* = 158; CD8 *n* = 150	Human tissue correlations; functional treatment experiments provide separate model evidence	TNC–autophagy/immune associations; p62-dependent TNC handling in models	No tissue mitophagy flux; TNC is non-mitochondrial cargo. No clinical validation of a p62-mitophagy checkpoint biomarker.
Evans et al. [45] Human expression component	Yes, tumor-derived RNA; 43 TNBCs; 14 recurrences	Pretreatment RNA-seq; randomized paclitaxel followed by FEC versus paclitaxel plus everolimus followed by FEC; arms pooled for expression–outcome analysis	OXPHOS expression associated with recurrence; separate residual-disease PDX experiments	No p62 IHC or flux measurement; expression associations do not validate a tissue-content/turnover panel.
Chourasia et al. [29] Human genomic/transcriptomic component	Human tumor datasets, not BNIP3 IHC; 113 TNBCs in CNV comparison and 101 in expression-survival analysis	TCGA copy-number analysis; separate pooled GEO expression-array survival analysis (101 TNBCs among 871 breast tumors)	*BNIP3* loss enriched in TNBC; low *BNIP3*/high *HIF1A* associated with poorer metastasis-free outcome	Human evidence is associative; causal evidence comes from mouse experiments. No FFPE panel validation.
Andrzejewski et al. [40] PROMIX expression reanalysis	Human tumor gene-expression data; mixed HER2-negative breast cancers	Baseline versus post-neoadjuvant tumor; epirubicin/docetaxel with response-guided bevacizumab in the parent trial	OXPHOS-gene enrichment in a subset of *MYC*-suppressed residual tumors across subtypes	Reanalysis of public data; not TNBC-only validation; no tissue measurements of mitochondrial flux, content or respiration.
Andrzejewski et al. [40] Paired BCL-XL IHC	Yes; 10 patients with breast cancer	Anthracycline-based neoadjuvant therapy; paired baseline/residual BCL-XL staining	Qualitative residual staining was similar to baseline	Survival-protein IHC only; mitochondrial turnover not measured.

Shading distinguishes headers and rows. Human tissue denotes clinical specimens and excludes xenografts. Study components and sample units are reported separately and cannot be combined into patient totals. None validates the proposed profiles. Andrzejewski’s PROMIX reanalysis and paired BCL-XL staining are separate components. Liu’s IHC and methylation analyses share the same 79 cases; the 42 TCGA cases are a subset of the 113. Abbreviations: TNBC, triple-negative breast cancer; p62, sequestosome 1 protein; IHC, immunohistochemistry; TMA, tissue microarray; DFS, disease-free survival; OS, overall survival; pCR, pathological complete response; AC, doxorubicin plus cyclophosphamide; COX-2, cyclooxygenase 2; PDX, patient-derived xenograft; METABRIC, Molecular Taxonomy of Breast Cancer International Consortium; RNA, ribonucleic acid; NRF2, nuclear factor erythroid 2-related factor 2; KEAP1, Kelch-like ECH-associated protein 1; TAC, docetaxel, doxorubicin and cyclophosphamide; p-NRF2, phosphorylated nuclear factor erythroid 2-related factor 2; MET, MET proto-oncogene receptor tyrosine kinase; TNFRSF19, tumor necrosis factor receptor superfamily member 19; FFPE, formalin-fixed, paraffin-embedded; TCGA, The Cancer Genome Atlas; ddAC, dose-dense doxorubicin plus cyclophosphamide; HIF1α/*HIF1A*, hypoxia-inducible factor 1 subunit alpha (protein/gene notation, respectively); LC3A, microtubule-associated protein 1 light chain 3 alpha; Bcl-2, B-cell lymphoma 2 protein; eIF2α, eukaryotic translation initiation factor 2 subunit alpha; TNC, tenascin-C; LC3B, microtubule-associated protein 1 light chain 3 beta; CD8, cluster of differentiation 8; RNA-seq, ribonucleic acid sequencing; FEC, fluorouracil, epirubicin and cyclophosphamide; OXPHOS, oxidative phosphorylation; *BNIP3*, B-cell lymphoma 2-interacting protein 3; CNV, copy-number variation; GEO, Gene Expression Omnibus; HER2, human epidermal growth factor receptor 2; *MYC*, MYC transcription factor; BCL-XL, B-cell lymphoma-extra-large protein; qMSP, quantitative methylation-specific polymerase chain reaction; PCR, polymerase chain reaction. PROMIX is the trial/dataset name; GSE22226 is a Gene Expression Omnibus series accession. ypT0 ypN0 denotes no residual primary tumor, including no in situ carcinoma, and no regional nodal metastasis after neoadjuvant treatment.

**Table 2:** Exploratory measurements and published reagents for FFPE TNBC.

Candidate Measurement	Compartment and Research Role	Published Reagent or Validation Status	Specific Scoring and Analytical Limitation
Total p62 [17,19,35,53,54]	Score tumor nuclear staining, diffuse cytoplasmic staining and puncta separately; distinguish stromal and immune cells	D-3, Santa Cruz, 1:100 in Luo; ab207305, Abcam, 1:2000 in Wang; ab56416, Abcam, 1:10,000 in Lee H. Published use does not establish interlaboratory TNBC qualification	Measure compartments separately; synthesis, degradation and condensates differ. Source cutoffs are not transferable.
LC3B [28,34]	Tumor and stromal puncta; autophagosome-associated material	No clone is recommended; assay-specific FFPE qualification required	Puncta density is sensitive to retrieval, sectioning and segmentation; staining does not distinguish formation from blockade.
p62 phospho-Ser403 [24,62]	Tumor cytoplasmic puncta; cargo-binding state	Exploratory extension; assay-specific FFPE qualification required	Phosphatase controls and an ischemia challenge required; not mitochondrial-specific and not proof of TBK1 activity.
TOMM20/TOM20 and COX IV [28,63]	Tumor cytoplasmic mitochondrial distribution; orthogonal content estimates	Exploratory pair; no cross-cohort TNBC assay standard endorsed	Normalize to viable tumor/cell size; selective protein loss and expression changes can uncouple staining from organelle mass.
4-HNE or 8-OHdG Author-proposed candidates	Cytoplasmic lipid damage or nuclear/cytoplasmic nucleic-acid damage, scored separately	Specificity, DNA/RNA discrimination, compartment and fixation controls required	Not mitochondrial-specific; mitochondrial attribution requires validated organelle colocalization.
NRF2 and KEAP1 [36,37]	Nuclear NRF2 and cytoplasmic KEAP1; redox-response context	Lee Y.S.: NRF2 sc-365949, KEAP1 sc-365626. Yu: phospho-NRF2 EP1809Y/ab76026, distinct from total staining. Taddei: NRF2 A-10/sc-365949 and MET D-4/sc-514148, each 1:100 [39]. Source cutoffs do not transfer.	Published cohorts do not validate the proposed panel; nuclear segmentation and application-specific specificity checks required.
LAMP1 [64]	Tumor lysosomal compartment; spatial context	Exploratory FFPE extension; functional findings in models do not qualify the tissue assay	Lysosome abundance does not measure acidity, proteolysis or mitochondrial delivery.
BNIP3 and NIX [29,30]	Mitochondrial receptor context; especially hypoxic regions	Candidate extensions; human *BNIP3* genomic data do not establish an FFPE clone	Expression has other functions; localization, receptor competence and delivery require testing in matched models.
OPTN and NDP52 [21]	Ubiquitin-dependent receptor recruitment; primarily assessed in models	Exploratory receptor measurements; assay-specific FFPE qualification required	Total staining cannot demonstrate recruitment to mitochondria; redundancy complicates negative results.
FUNDC1 [31]	Hypoxia-regulated mitochondrial receptor, mechanism-specific extension	Primary receptor studies are non-TNBC; exploratory FFPE measurement	Abundance does not report regulatory state, LC3 engagement or turnover.
PINK1 and Parkin [19,20,21]	Damage recognition and recruitment in models; exploratory tissue context	Parkin sc-32282, 1:50 in Wang; no qualified PINK1 stain endorsed	Diffuse expression does not establish mitochondrial stabilization/translocation; alternative routes can bypass the pathway.
DRP1 and OPA1 [19,63]	Dynamics/cristae context; mitochondrial recruitment or processing assessed in models	DRP1 ab56788, 1:1000 in Wang; assay-specific FFPE qualification required for the proposed combination	Total protein measurements do not assess fission, long/short OPA1 processing or respiratory dependence.

Shading distinguishes headers and rows. The proposed combinations lack analytical and clinical validation. Reagents are listed for traceability and require application- and lot-specific qualification. Abbreviations: FFPE, formalin-fixed, paraffin-embedded; TNBC, triple-negative breast cancer; p62, sequestosome 1 protein; LC3, microtubule-associated protein 1 light chain 3; LC3B, microtubule-associated protein 1 light chain 3 beta; Ser403, serine residue 403 of p62; TBK1, TANK-binding kinase 1; TANK, tumor necrosis factor receptor-associated factor family member-associated nuclear factor kappa-B activator; TOMM20/TOM20, translocase of outer mitochondrial membrane 20; COX IV, cytochrome c oxidase subunit IV; 4-HNE, 4-hydroxy-2-nonenal; 8-OHdG, 8-hydroxy-2′-deoxyguanosine; DNA, deoxyribonucleic acid; RNA, ribonucleic acid; NRF2, nuclear factor erythroid 2-related factor 2; KEAP1, Kelch-like ECH-associated protein 1; phospho-NRF2, phosphorylated nuclear factor erythroid 2-related factor 2; MET, MET proto-oncogene receptor tyrosine kinase; LAMP1, lysosome-associated membrane protein 1; BNIP3, B-cell lymphoma 2-interacting protein 3; NIX, B-cell lymphoma 2-interacting protein 3-like protein; OPTN, optineurin; NDP52, nuclear dot protein 52; FUNDC1, FUN14 domain-containing protein 1; PINK1, PTEN-induced kinase 1; PTEN, phosphatase and tensin homolog; Parkin, parkin ubiquitin ligase (protein name); DRP1, dynamin-related protein 1; OPA1, optic atrophy 1 mitochondrial dynamin-like protein.

**Table 3:** Minimum definitions of candidate tissue profiles for research.

Descriptive Tissue Profile	Expected Registered Tumor Region Measurements	Hypothesized Dependency and Required Functional Result	Findings that Challenge or Falsify the Interpretation
A. Damage with preserved/enriched content and redox signaling	TOMM20 and COX IV each preserved or increased; 4-HNE or 8-OHdG increased; nuclear NRF2 increased. Record cytoplasmic p62. Phospho-p62 Ser403 is an optional extension once qualified; neither p62 readout is required to be high. Attributing damage to mitochondria requires colocalization.	Candidate redox/respiratory adaptation: matched-model ROS and OCR measurements; selective redox or respiratory perturbation impairs survival/regrowth with appropriate rescue.	Damage is shown to be nuclear/non-mitochondrial only; preserved/increased-content criterion fails after size/cellularity correction; the model has neither redox nor respiratory dependency despite adequate perturbation.
B. Damage with cargo/lysosome-associated accumulation	Cytoplasmic p62 puncta and LC3B puncta increased in the same region; LAMP1 context documented; 4-HNE or 8-OHdG increased; content preserved/increased on the prespecified qualified TOMM20 or COX IV assay. When both markers are measured, content is indeterminate if one is reduced and the other is preserved or increased. OPTN/NDP52 or BNIP3/NIX are optional context-dependent extensions.	Two competing mechanisms: adaptive delivery or impaired downstream disposal. Distinguish them using reporter kinetics and lysosomal/cargo turnover controls; test autophagy dependence separately.	Impaired downstream disposal explains accumulation, challenging an efficient-clearance interpretation; no mitochondrial cargo delivery; p62 staining fails the required cytoplasmic-puncta criterion or reflects non-mitochondrial cargo; selective delivery inhibition does not alter the proposed dependency.
C. Damage with reduced mitochondrial content	TOMM20 and COX IV decreased beyond repeatability limits after normalization to viable carcinoma; damage increased. The model does not constrain p62/Ser403/LC3B directions; LAMP1 and receptor measurements provide context.	Candidate clearance-driven organelle loss: demonstrate increased delivery and independent cargo turnover; exclude reduced biogenesis/cell dilution. A lethal-clearance interpretation requires evidence that specific blockade preserves mass, OCR and clonogenic survival under comparable initial injury.	Low content explained by cell size, necrosis, reduced biogenesis or direct drug injury; no increased delivery. For the lethal-clearance interpretation: death precedes organelle loss; effective clearance blockade fails to rescue mass, respiration and viability.

Shading distinguishes headers and rows. These profiles can overlap and have no validated TNBC thresholds or established role in treatment selection. Marker directions refer to a reference fixed during training or a matched baseline; a paired change qualifies only if it exceeds the assay-specific 95% repeatability limit. Assignment requires the defining criteria to be met together, subject to the stated alternatives; optional measurements do not determine classification. Profile A requires both TOMM20 and COX IV to be preserved or increased, and profile C requires both to be decreased. Content is indeterminate when one marker is reduced and the other is preserved or increased; assignment then awaits orthogonal assessment. Profile B retains competing explanations until dynamic testing distinguishes them. Abbreviations: TNBC, triple-negative breast cancer; TOMM20, translocase of outer mitochondrial membrane 20; COX IV, cytochrome c oxidase subunit IV; 4-HNE, 4-hydroxy-2-nonenal; 8-OHdG, 8-hydroxy-2′-deoxyguanosine; NRF2, nuclear factor erythroid 2-related factor 2; p62, sequestosome 1 protein; Ser403, serine residue 403 of p62; ROS, reactive oxygen species; OCR, oxygen-consumption rate; LC3B, microtubule-associated protein 1 light chain 3 beta; LAMP1, lysosome-associated membrane protein 1; OPTN, optineurin; NDP52, nuclear dot protein 52; BNIP3, B-cell lymphoma 2-interacting protein 3; NIX, B-cell lymphoma 2-interacting protein 3-like protein.

**Table 4:** Selected preclinical experiments relevant to mitochondrial stress in TNBC.

Evidence Level and Study	Model/Sample Unit	Exposure and Context	Endpoint	Mitochondrial Delivery or Flux	Causal Perturbation and Validation Limit
Patient-derived models Evans [45]	10 TNBC PDXs: 8 post-neoadjuvant residual tumors and 2 tumors from donors without neoadjuvant therapy; initial screening cohorts of 2–4 mice/model	IACS-10759 complex I inhibition: 5 mg/kg orally, 5 days on/2 days off; donor treatment histories differ	Tumor response and expression-associated respiratory sensitivity	Not a mitophagy-flux study	Respiratory pharmacology; separate human RNA component (*n* = 43) in Table 1.
Patient-derived models Berner [51]	2 PDX models; one growth experiment *n* = 6 mice/arm; 3750 mitochondria/800 lipid droplets are nested counts	AC, docetaxel, carboplatin or combinations; residual state	3D mitochondrial architecture and lipid-droplet contacts	Static ultrastructure, not flux	Descriptive treatment comparison; no clearance-specific rescue.
Patient-derived models Lee H. [35]	37 TNBC PDX tumors	Donor tissue collected 2015–2020	COX-2–p62 association in PDX tumors	Static IHC; no mitochondrial flux measurement	No causal perturbation; separate clinical component in Table 1.
Patient-derived/cell models Baek [63]	PIM001-P TNBC PDX and TNBC lines; post-AC TEM, *n* = 3 mice/group; MYLS22 residual-regrowth experiment, *n* = 6 mice/arm	PDX: single intraperitoneal AC (doxorubicin 0.5 mg/kg; cyclophosphamide 50 mg/kg); MYLS22 every 2 days from day 21. MDA-MB-231: doxorubicin 100 nM, then MYLS22 50 µM 48 h later.	OPA1-linked mitochondrial architecture, content and respiration; time to residual-tumor regrowth	Not a mitophagy-flux study	Genetic perturbation of *OPA1* and pharmacological inhibition; no clearance-specific rescue. Total OPA1 IHC does not resolve processing.
Patient-derived/cell models Jiang [67]	Three TNBC PDX models from two donors (PIM001-P and PIM001-M from one donor; PIM005 from another); single-cell RNA sequencing of nine tumor samples. Functional studies in HCC1143 and HCC1937 cells.	Single intraperitoneal AC dose (doxorubicin 0.5 mg/kg plus cyclophosphamide 50 mg/kg); residual tumors at day 21 and regrown tumors at day 40. Cell experiments used doxorubicin and phosphoramide mustard.	*C15ORF48*-enriched basal cells and ROS buffering	Not a mitophagy-flux study	C15ORF48 depletion increased ROS/apoptosis and sensitization; p62 dependence not established.
Patient-derived/cell models Taddei [39]	3 TNBC PDO cultures (21, 43, 46), plus murine/human cell models	Paclitaxel with PHA-665752 (MET), dasatinib (SRC) or ML-385 (NRF2); PDO endpoint after 5 days of treatment	Viability, chemosensitivity and redox signaling	Not a mitophagy-flux study	Orthogonal pathway interventions.
Cell-line resistance Uddin [77]	SUM159 and MDA-MB-468 parental/resistant pairs; SUM159 proteomics *n* = 5 biological replicates/group	Olaparib resistance acquired over 6 months of selection; both pairs *BRCA1/2*-intact; MDA-MB-468 HR-defective	Lower p62; olaparib sensitivity	No mitochondrial-selective reporter; general-autophagy interpretation based on steady-state measurements	siRNA-mediated p62 depletion; clearance-specific rescue not demonstrated.
Cell-line resistance Hong [78]	Parental and PARP-inhibitor-resistant MDA-MB-436 cells (*BRCA1*-mutant); combination growth assays, *n* = 3 independent experiments	Nitro-oleic acid (0.3–5 µM) with graded olaparib or talazoparib; dosing on days 1 and 4, viability on day 7	Growth inhibition and PARP-inhibitor resensitization; p62 alkylation and lysosomal adaptation	Companion assays: LC3 turnover with bafilomycin A1 and mCherry–EGFP–LC3B reporter; general autophagy, not mitochondrial-selective flux.	p62 knockdown in TNBC; wild-type/Cys105/113-mutant p62 reconstitution in mouse fibroblasts. Multi-target electrophilic pharmacology; no tissue-profile validation.
Cell culture/xenograft Chatterjee [64]	MDA-MB-231 and Hs 578T cultures in 2D/3D; LAMP1-depletion colony assays in MDA-MB-231, *n* = 3 independent experiments; MDA-MB-231 xenografts	Doxorubicin- or paclitaxel-tolerant states generated over 12 days; autophagy and lysosomal interventions	Persister survival and regrowth	Bulk autophagy/lysosomal effects	LAMP1 depletion; broad lysosomal dependence does not establish selective mitophagy.
Cell culture/syngeneic mouse Ho [85]	Five TNBC cell lines; two-drug cytotoxicity assays, *n* = 3–4 experiments per line; metM-Wnt^lung^ syngeneic tumor study (120 mice in the animal-study cohort)	Carboplatin with hydroxychloroquine and/or BMS-754807. In mice: carboplatin 50 mg/kg weekly, hydroxychloroquine 60 mg/kg and BMS-754807 6.25 mg/kg daily; intraperitoneal administration	Viability and tumor response	Bulk autophagy measures; selective mitochondrial flux not established	ATG5 depletion left BMS-754807/carboplatin sensitivity unchanged; HCQ effects were not explained by autophagy inhibition alone.
PDO/cell/xenograft models Andrzejewski [40]	MDA-MB-231, SUM159 and HCC1806, with MDA-MB-468 as a contrasting model; one TNBC PDO line, HCI-002. Cell assay results reported from ≥3 independent experiments; bilateral MDA-MB-231 xenografts	Cell-persistence protocol: doxorubicin 150 or 250 nM versus docetaxel 15 nM for 2 h, followed by washout. Mice: doxorubicin 3 mg/kg and/or DT2216 15 mg/kg intraperitoneally twice weekly for 6 weeks; first DT2216 dose 3 days after doxorubicin in the combination arm.	OXPHOS-high persistence, BCL-XL survival dependency, viability and tumor response	Not a mitophagy-flux study	CRISPR-mediated BCL-XL depletion in cell lines; BCL-XL inhibitor response in HCI-002; inhibitors/degrader in other models. Dependency varied by model/exposure. Human components in Table 1.
Cell culture/xenograft Liu [41]	MDA-MB-231 and BT-549; selected MDA-MB-231 doxorubicin-response xenograft, *n* = 5 mice/group	*TNFRSF19* gain/loss with doxorubicin; selected xenografts received 3 mg/kg intraperitoneally once weekly	DNA damage, apoptosis and doxorubicin sensitivity	TEM, cargo markers and LC3B–TOM20 colocalization; tandem LC3B reporter measures bulk autophagy, not selective mitochondrial flux	*TNFRSF19* perturbation and CCCP/Mdivi-1 reversal; limited pharmacological specificity. Human data in Table 1.
Cell culture Chu [62]	MDA-MB-231 and 4T1 cells; selected mt-Keima and TEM assays in MDA-MB-231, reported *n* = 3 per assay.	Bi-magnolignan for 24 h: 20 µM in MDA-MB-231 and 8 µM in 4T1; BX795 co-treatment at 1 and 0.5 µM, respectively.	Depolarization, mitochondrial content and DNA, mitochondrial proteins, viability and apoptosis	mt-Keima acidic-compartment signal and TEM; chloroquine-sensitive mitochondrial loss. Reporter responses to chloroquine are also sensitive to lysosomal pH.	*TBK1* knockout, BX795 and chloroquine attenuated selected mitochondrial changes or death. TBK1 pleiotropy and broad autophagy inhibition limit attribution to selective mitochondrial clearance.
Cell culture Li [71]	MDA-MB-231; selected mitochondrial-colocalization assay: 3 independent experiments, 30 cells per experiment.	Polyphyllin I, 8 µM for 6 h in the PINK1-depletion comparison; separate lysosomal-blockade experiment: 2 µM for 9 h ± bafilomycin A1 (25 nM).	Mitochondrial injury, clearance-associated changes and apoptosis	Mitochondrial–LC3–LAMP1 colocalization and mitochondrial protein loss; bafilomycin A1 attenuated TOMM20 loss in a separate experiment.	PINK1 depletion reduced clearance-associated readouts but increased apoptosis, challenging a simple lethal-clearance interpretation.
Cell culture/xenograft drug conjugates Liu [87]	MDA-MB-231 cells; apoptosis assay, reported *n* = 3. MDA-MB-231 xenografts: tumor-growth and survival analyses, each reported as *n* = 5 mice/group; terminal tumor weights, *n* = 3/group.	Paclitaxel/2-methoxyestradiol conjugates; 48-h apoptosis assays. Survival experiment: tail-vein dosing every 3 days, starting at a tumor volume of approximately 80 mm^3^.	Mitochondrial morphology, apoptosis, tumor growth and survival	MitoTracker morphology, TEM and static PINK1/Parkin, p62 and LC3 measurements; no mitochondrial-selective flux reporter.	3-Methyladenine and Mdivi-1 reduced apoptosis. Their broad effects do not establish selective clearance-dependent death; no genetic clearance-specific rescue.
Cell/immune/animal models Li [44]	4T1 syngeneic tumors in BALB/c mice; anti-PD-1 experiments, *n* = 6 mice/arm; anti-PD-L1 experiments, *n* = 8 mice/arm. Human component in Table 1.	Autophagy-deficient versus wild-type tumors. Anti-PD-1 (29F.1A12), 100 µg intraperitoneally on days 6, 10 and 14; anti-PD-L1 (10F.9G2), 100 µg intraperitoneally on days 5 and 10 after inoculation.	T-cell cytotoxicity and tumor response; tumor weights at day 18 (PD-1) or day 14 (PD-L1)	Bulk autophagy and TNC degradation, not mitophagy	ATG5/Beclin-1 and TNC/p62-related perturbations; TNC deletion restored T-cell killing in autophagy-deficient models, supporting a non-mitochondrial mechanism.
Patient-derived/cell radiotherapy models Huang [82]	MC1 TNBC PDX and HCC1806 genetic xenograft: separate experiments, *n* = 4 mice/group; TNBC cells	MC1: PC3-15 50 mg/kg ± irradiation (3 Gy × 4). HCC1806 genetic experiment: 4 Gy × 5	DNA repair, survival and radiation response	General autophagy; mitochondrial-selective flux not established	*HECTD3* knockout and p62 knockdown/rescue support DNA repair/radiosensitivity.
Cell radiotherapy/orthotopic mouse tumor models Yadav [83]	Parental/radioresistant MDA-MB-231 cells; autophagy-blockade clonogenic assays, 3 wells/group. 4T1-luc BALB/c allograft growth: *n* = 7 mice/group.	Cell X-rays, 0.5–4 Gy; colonies at 14 days. Separate non-irradiated mice: XRK3F2, 27 mg/kg/day intraperitoneally, 5 days/week for 2 weeks.	Autophagy and radiation survival in cells; tumor growth in mice	LC3 turnover with chloroquine or bafilomycin A1; general autophagic flux, not mitochondrial-selective flux	*ATG5* knockout/chloroquine for radiation response; p62 depletion/inhibition for growth and VCAM-1 expression. No irradiated-mouse validation.
Cell/mouse metabolic targeting Shen [86]	Primarily 4T1 and tumor-bearing mice; respiratory assays *n* ≥ 3 independent samples	Epirubicin plus mitochondria-targeted lonidamine delivery	Respiration, ATP and tumor response	Not a mitophagy-flux study	Metabolic pharmacology; no selective clearance perturbation.

Shading distinguishes headers and rows. Clinical evidence is summarized separately in Table 1. Sample sizes refer to the specified experiments; reported *n* does not necessarily denote independent biological replicates. Wells, cells and organelles are distinguished from independent cultures or animals. For MYLS22, Baek et al. report 10 mg/kg by peritumoral injection in their Methods and 100 mg/kg by intratumoral injection in the legend to their Figure 7A [63]. Abbreviations: TNBC, triple-negative breast cancer; PDX, patient-derived xenograft; AC, doxorubicin plus cyclophosphamide; 3D, three-dimensional; COX-2, cyclooxygenase 2; p62, sequestosome 1 protein; IHC, immunohistochemistry; LAMP1, lysosome-associated membrane protein 1; TEM, transmission electron microscopy; OPA1, optic atrophy 1 mitochondrial dynamin-like protein; *C15ORF48*, chromosome 15 open reading frame 48; ROS, reactive oxygen species; PDO, patient-derived organoid; MET, MET proto-oncogene receptor tyrosine kinase; SRC, SRC proto-oncogene non-receptor tyrosine kinase; NRF2, nuclear factor erythroid 2-related factor 2; *BRCA1/2*, breast cancer susceptibility genes 1 and 2; HR, homologous recombination; siRNA, small interfering ribonucleic acid; PARP, poly(adenosine diphosphate-ribose) polymerase; 2D, two-dimensional; ATG5, autophagy-related protein 5; HCQ, hydroxychloroquine; OXPHOS, oxidative phosphorylation; BCL-XL, B-cell lymphoma-extra-large protein; CRISPR, clustered regularly interspaced short palindromic repeats; TNFRSF19, tumor necrosis factor receptor superfamily member 19; DNA, deoxyribonucleic acid; LC3, microtubule-associated protein 1 light chain 3; LC3B, microtubule-associated protein 1 light chain 3 beta; TOM20/TOMM20, translocase of outer mitochondrial membrane 20; CCCP, carbonyl cyanide 3-chlorophenylhydrazone; PINK1, PTEN-induced kinase 1; PTEN, phosphatase and tensin homolog; TNC, tenascin-C; PD-1, programmed cell death protein 1; PD-L1, programmed death-ligand 1; ATG, autophagy-related protein; HECTD3, HECT-domain ubiquitin-protein ligase 3 (HECT, homologous to the E6-associated protein carboxyl terminus); VCAM-1, vascular cell adhesion molecule 1; ATP, adenosine triphosphate; RNA, ribonucleic acid; mt-Keima, mitochondria-targeted Keima fluorescent reporter; TBK1, TANK-binding kinase 1; TANK, tumor necrosis factor receptor-associated factor family member-associated nuclear factor kappa-B activator; EGFP, enhanced green fluorescent protein; Cys, cysteine; pH, measure of acidity defined by the negative base-10 logarithm of hydrogen-ion activity; Gy, gray; h, hour.

## Data Availability

No new patient-level datasets were generated or analyzed. Source publications and the evidence-appraisal framework are identified in the manuscript and Supplementary Materials.

## Supplementary Materials

The following supporting information can be downloaded at: https://www.mdpi.com/article/10.3390/cancers18213589/s1, Table S1: Review scope and evidence appraisal; Table S2: Evidence supporting the figures and limits of interpretation; Table S3: Evidence counts by type for selected claims in this review. References [1,2,6,11,12,13,14,15,16,17,18,19,20,21,22,23,24,25,26,27,28,29,30,31,34,35,36,37,38,39,40,41,44,45,51,55,56,57,58,63,65,67] are cited in the Supplementary Materials.

## Abbreviations

The following abbreviations are used in this manuscript:

2D/3D	two-dimensional/three-dimensional
4-HNE	4-hydroxynonenal
8-OHdG	8-hydroxy-2′-deoxyguanosine
AC	doxorubicin and cyclophosphamide
aPKC	atypical protein kinase C
ASCO/CAP	American Society of Clinical Oncology/College of American Pathologists
ATG	autophagy-related
ATG5 and ATG7	autophagy-related proteins 5 and 7
ATP	adenosine triphosphate
Bcl-2	B-cell lymphoma 2 protein
BCL-XL	B-cell lymphoma-extra large
BNIP3	BCL2 interacting protein 3
*BRCA1/2*	breast cancer susceptibility genes 1 and 2
*C15ORF48*	chromosome 15 open reading frame 48
CALCOCO2	calcium binding and coiled-coil domain 2
CaMKII	calcium/calmodulin-dependent protein kinase II
CCCP	carbonyl cyanide m-chlorophenyl hydrazone
CD8	cluster of differentiation 8
CNV	copy number variation
COX-2	cyclooxygenase-2
COX IV	cytochrome c oxidase subunit IV
CRISPR	clustered regularly interspaced short palindromic repeats
Cys	cysteine
ddAC	dose-dense doxorubicin and cyclophosphamide
DFS	disease-free survival
DNA	deoxyribonucleic acid
DRP1	dynamin-related protein 1
EGFP	enhanced green fluorescent protein
eIF2α	eukaryotic translation initiation factor 2 subunit alpha
EMT	epithelial–mesenchymal transition
ER	estrogen receptor
ERK1/2	extracellular signal-regulated kinases 1 and 2
FEC	Fluorouracil, epirubicin and cyclophosphamide
FFPE	formalin-fixed paraffin-embedded
FUNDC1	FUN14 domain-containing protein 1
GABARAP	gamma-aminobutyric acid type A receptor-associated protein
GATA3	GATA-binding protein 3
GEO	Gene Expression Omnibus
H&E	hematoxylin and eosin
HCQ	hydroxychloroquine
HECTD3	HECT-domain E3 ubiquitin-protein ligase 3 (HECT, homologous to the E6-associated protein carboxyl terminus)
HER2	human epidermal growth factor receptor 2
*HIF1A*/HIF1α	hypoxia-inducible factor 1 alpha gene/protein
HR	homologous recombination
HRD	homologous recombination deficiency
ICC	intraclass correlation coefficient
ICI	immune checkpoint inhibitor
IHC	immunohistochemistry
IKK	IκB kinase (IκB, inhibitor of nuclear factor κB)
ITCH	itchy E3 ubiquitin protein ligase
KEAP1	Kelch-like ECH-associated protein 1
KIR	KEAP1-interacting region
LAMP1	lysosome-associated membrane protein 1
LC3	microtubule-associated protein 1 light chain 3; LC3A and LC3B, microtubule-associated protein 1 light chain 3 alpha and beta, respectively
LIR	LC3-interacting region
MET	MET receptor tyrosine kinase
METABRIC	Molecular Taxonomy of Breast Cancer International Consortium
mito-QC	mitochondrial quality-control fluorescent reporter
mtDNA	mitochondrial DNA
mt-Keima	mitochondria-targeted Keima fluorescent reporter
mTORC1	mechanistic target of rapamycin complex 1
NBR1	NBR1 autophagy cargo receptor
NDP52	nuclear dot protein 52/CALCOCO2
NF-κB	nuclear factor κB
NIX/BNIP3L	B-cell lymphoma 2-interacting protein 3-like protein
NRF2	nuclear factor erythroid 2-related factor 2; p-NRF2/phospho-NRF2, phosphorylated NRF2
OCR	oxygen-consumption rate
OPA1	optic atrophy 1 mitochondrial dynamin-like protein
OPTN	optineurin
OS	overall survival
OXPHOS	oxidative phosphorylation
PARP	poly(adenosine diphosphate-ribose) polymerase
PB1	Phox and Bem1p domain
pCR	pathological complete response
PCR	polymerase chain reaction
PD-1	programmed cell death protein 1
PD-L1	programmed death-ligand 1
PDO	patient-derived organoid
PDX	patient-derived xenograft
pH	measure of acidity defined by the negative base-10 logarithm of hydrogen-ion activity
PINK1	PTEN-induced kinase 1 (PTEN, phosphatase and tensin homolog)
PR	progesterone receptor
*PRKN*	Parkin E3 ubiquitin-protein ligase gene
qMSP	quantitative methylation-specific polymerase chain reaction
Rag GTPases	Ras-related guanosine triphosphate (GTP)-binding proteins
Raptor	regulatory-associated protein of mTOR
RCB	residual cancer burden
RNA	ribonucleic acid
RNA-seq	RNA sequencing
ROS	reactive oxygen species
SCAMP3	secretory carrier membrane protein 3
Ser403	serine residue 403 of p62
siRNA	small interfering ribonucleic acid
SQSTM1/p62	sequestosome 1
SRC	SRC non-receptor tyrosine kinase
TAC	Docetaxel, doxorubicin and cyclophosphamide
TBK1	TANK-binding kinase 1 (TANK, tumor necrosis factor receptor-associated factor family member-associated nuclear factor kappa-B activator)
TCGA	The Cancer Genome Atlas
TEM	transmission electron microscopy
TIL	tumor-infiltrating lymphocyte
TMA	tissue microarray
TMRE	tetramethylrhodamine ethyl ester
TMRM	tetramethylrhodamine methyl ester
TNBC	triple-negative breast cancer
TNC	tenascin-C
TNFRSF19	tumor necrosis factor receptor superfamily member 19
TOMM20/TOM20	translocase of outer mitochondrial membrane 20
TRAF6	tumor necrosis factor receptor-associated factor 6
TRPML1	transient receptor potential mucolipin 1
TRPS1	transcriptional repressor GATA binding 1
Ub	ubiquitin
UBA	ubiquitin-associated domain
UTR	untranslated region
VCAM-1	vascular cell adhesion molecule 1
ZZ	ZZ-type zinc-finger domain
