# Supplementary material for: SQSTM1/p62 and Mitochondrial Stress in Triple-Negative Breast Cancer: A Critical Pathology Perspective
Source: Cancers (Basel). 2026 Nov 9;18(21):3589. doi: 10.3390/cancers18213589 (PMC13761476; doi:10.3390/cancers18213589)
Supplement: Supplementary file 1 [file cancers-18-03589-s001.zip › cancers-4665762-supplementary.pdf]

Supplementary Materials

Supplementary Table S1. Review scope and evidence appraisal. Part A. Literature discovery and selection.

Element	Approach
Review scope and date	This selective narrative review considered literature available through 1 November 2026. No review protocol was registered.
Discovery route	Sources were identified through web searches restricted to PubMed-indexed records, followed by inspection of PubMed records or original publications and citation tracing. These were targeted web searches, rather than queries run through the PubMed search engine; no complete database export was produced.
p62 in human TNBC	site:pubmed.ncbi.nlm.nih.gov (SQSTM1 OR p62) ("triple-negative breast cancer" OR TNBC) (immunohistochemistry OR tissue OR neoadjuvant)
Mitophagy receptors	site:pubmed.ncbi.nlm.nih.gov (BNIP3 OR NIX OR FUNDC1 OR OPTN OR NDP52) ("breast cancer" OR mitophagy)
Mitochondrial reporters	site:pubmed.ncbi.nlm.nih.gov ("mt-Keima" OR "mito-QC") (mitophagy OR lysosomal)
Companion tissue markers	site:pubmed.ncbi.nlm.nih.gov (NRF2 OR KEAP1 OR autophagy) "triple-negative" (immunohistochemistry OR neoadjuvant)
Further source checks	Searches by DOI, study title and author were used to check primary methods and figure captions for the main human, experimental and assay claims. Citation tracing included original studies and recent reviews. Additional checks on 1 November 2026 addressed LC3 isoforms and staining patterns, variation among biopsies from the same patient, and histological types and diagnostic mimics in TNBC. Full-text reports and associated supplementary material were checked again on 2 November 2026 to align experimental sample units, exposures and assay interpretations.
Reports within scope	Reports were selected for their relevance to p62 or companion markers in human TNBC, TNBC pathology and diagnostic context, mechanisms of mitochondrial quality control, or original assay methodology. Findings from other cancers were considered only when they addressed a defined measurement problem, with their indirect relevance stated.
Reports outside scope	Reports were outside scope when they concerned unrelated organ-specific biology, generic angiogenesis or imaging without a defined measurement role, redundant reviews of biomarkers in other cancers, or claims without a direct link to the subject.
Language and selection	English-language reports were considered; systematic translation was not undertaken. Sources were assessed selectively, without dual independent screening. Reviews provided background and were not counted as additional primary cohorts.
Duplicates and exclusions	Each publication has one bibliography entry. Its clinical and experimental components are identified separately but are not treated as independent studies. Complete retrieval, duplicate-removal and exclusion logs were not maintained for this selective review.
Bibliography and evidence counts	The manuscript cites 97 publications. This bibliography count is not the number of retrieved or eligible studies. Supplementary Table S3 inventories only selected evidence components for named claims; its counts do not represent pooled patient numbers or complete coverage of the literature.

The search formulations describe how sources were identified for selective review and appraisal; they do not constitute an exhaustive search of the PubMed database.

Abbreviations: DOI, digital object identifier; p62, sequestosome 1 protein; SQSTM1, sequestosome 1 (gene/protein designation); TNBC, triple-negative breast cancer; BNIP3, B-cell lymphoma 2-interacting protein 3; NIX, B-cell lymphoma 2-interacting protein 3-like protein; FUNDC1, FUN14 domain-containing protein 1; OPTN, optineurin; NDP52, nuclear dot protein 52; mt-Keima, mitochondria-targeted Keima fluorescent reporter (reporter name); mito-QC, mitochondrial quality-control fluorescent reporter (reporter name); NRF2, nuclear factor erythroid 2-related factor 2; KEAP1, Kelch-like ECH-associated protein 1; LC3, microtubule-associated protein 1 light chain 3.

Supplementary Table S1 (continued). Part B: Dimensions used for qualitative appraisal of the evidence.

Dimension	What the appraisal considers	Limits on interpretation
Population and specimen directness	Clinical TNBC tissue is distinguished from mixed breast cohorts with a stated TNBC denominator, patient-derived and conventional models, human expression datasets, and methodological examples.	Patient-derived xenografts provide model tissue. Findings pooled across breast-cancer subtypes do not establish a TNBC-specific association; mixed cohorts require separately reported TNBC analyses. Findings from other tumor types remain indirect.
Exposure and timing	The exact regimen, setting, prior exposure, sampling time and *BRCA*/HRD context are considered.	Findings from different therapies or time points cannot be treated as interchangeable. An association measured after treatment does not provide baseline treatment prediction.
Analytical validity	Assay assessment covers the antibody or other reagent and its controls, the compartment and scoring method, repeatability, and sensitivity to pre-analytical conditions.	Use in a published study does not establish analytical validity. A stain that lacks the required qualification remains a candidate measurement.
Dynamics	Time-resolved measurements of mitochondrial delivery or turnover are distinguished from bulk-autophagy reporters, static markers and ultrastructural evidence.	Static staining and bulk autophagy do not establish selective mitochondrial flux. Reporter interpretation depends on pH and the available dynamic range.
Causality	Causal evidence is assessed through specific genetic loss, an independent targeting reagent, functional endpoints, and rescue with wild-type and informative-mutant constructs.	A drug effect or the co-occurrence of markers does not, on its own, identify a dependency on clearance. Perturbing a pleiotropic node requires additional experiments to distinguish clearance-specific effects from its other activities.
Statistical support	The biological unit, sample size (n), endpoint, effect estimate and uncertainty are considered, together with adjustment, multiplicity and model validation.	Organelles and regions are nested measurements, not independent patients. Statistical significance alone does not validate a risk factor; interpretation also depends on effect size, uncertainty, confounding, multiplicity and independent validation.
Independent validation	The appraisal examines whether a locked assay or profile has been tested in a distinct cohort or model and considers the independence of the patients and datasets.	Transcriptomic corroboration does not validate an IHC assay. Repeating model experiments within a single publication does not provide independent clinical validation.
Qualitative interpretation	The narrative appraisal links observed findings to the claims they support and distinguishes clinical associations from experimental mechanisms and proposed hypotheses.	These distinctions guide the narrative interpretation; they do not constitute a complete per-study categorical matrix or a numerical total score. Clinical directness and mechanistic strength remain separate.
Selection boundaries	Inclusion requires a defined, relevant claim. Reviews provide background; cross-cancer examples are included when they address an explicit measurement question.	Each source needs a stated role in the subject or in the measurement approach. General examples from unrelated diseases remain outside scope.

Abbreviations: TNBC, triple-negative breast cancer; *BRCA*, breast cancer susceptibility genes 1 and 2; HRD, homologous recombination deficiency; pH, measure of acidity defined by the negative base-10 logarithm of hydrogen-ion activity; IHC, immunohistochemistry.

Supplementary Table S2. Evidence supporting the figures and limits of interpretation.

Figure connection or element	Evidence and experimental context	Interpretation and limits
1: selected p62 module strip	Structural reviews [14,15,16]; primary PB1/UBA and LC3 experiments [23,24]	The strip shows selected domains and binding regions in sequence order, without quantitative lengths or residue positions. Raptor/Rag binding sites are not assigned.
1 panel 1: cargo–p62–LC3 engagement	Cargo condensation, ubiquitin handling and LC3 incorporation in primary experiments [23,24]; ATG8-family context [14,15,16]	The scene depicts cargo and LC3 engagement. Establishing productive autophagy or selective mitochondrial flux requires additional evidence.
1 panel 2: KEAP1–NRF2 regulation	Phosphorylation-dependent p62–KEAP1 binding and NRF2 regulation [22]	Binding of p62 to KEAP1 can indirectly stabilize NRF2; the figure does not depict direct p62–NRF2 binding.
1 panel 3: NF-κB pathway	Primary IL-1/TRAF6 experiments examining p62 binding and aPKC/IKK involvement [26]	The simplified pathway summarizes signaling in non-TNBC models, without implying direct enzymatic action by p62 or a dependency in human tumors.
1 panel 4: nutrient sensing / mTORC1	Separate p62–Raptor/Rag interactions and amino-acid-dependent lysosomal recruitment [25]	Rag lies outside the mTORC1 core; its binding is not mapped to the region that interacts with Raptor.
1 panel 5: ubiquitin / proteostasis	Primary condensation, ubiquitin handling and phosphorylation-dependent processing [23,24,27]	Condensates organize cargo. p62 abundance alone cannot establish degradation, mitochondrial specificity or a functional dependency.
1: TNBC context	Clinical and research context [1,2,6]; molecular studies listed above	The TNBC context does not establish that these pathways operate in individual human tumors.
1 caption: mitochondrial clustering and alternative receptors	Clustering and degradation experiments in Parkin models [20]; alternative receptor mechanisms [21,29,30,31]	Clustering alone cannot establish that p62 is required for mitochondrial degradation. The receptor routes are alternatives, not one p62-dependent cascade.
2: high/low p62 alternatives	Synthesis, phosphorylation, condensation and degradation [22,23,24,27,28,55]	Several processes can produce high or low p62 measurements; low p62 alone cannot demonstrate mitochondrial clearance.
2: independent functional evidence	Autophagy guidance, receptor experiments and mitochondrial reporters [20,21,28,29,30,31,56,57,58]	Dynamic and orthogonal measurements test the competing explanations; static markers and bulk-autophagy reporters cannot establish selective mitochondrial flux.
2: tissue/compartment context	Human observations [17,18,19,34,35]; cohort evidence summarized in Table 1 and Table S3	The panels outline possibilities for tissue interpretation, rather than findings from the cited cohorts.
3: clinical branches and profiles	Endpoint and reporting standards [11,12,13,65]; proposed research profiles in Table 3	Arrows indicate study sequence; pCR corresponds to RCB 0. Paired analysis requires viable carcinoma from the same anatomical compartment. Patients with residual disease confined to lymph nodes cannot contribute a breast paired-change measurement. Table 3 defines marker directions and joint or alternative criteria for the research profiles. Content is indeterminate when one mitochondrial-content marker is reduced and the other is preserved or increased.
4 A–C: functional testing	Assay guidance, receptor experiments and engineered reporters [20,21,28,29,30,31,56,57,58]	The panels distinguish measurements, selective loss-of-function experiments with rescue, and conditional predictions. Testing adaptive or death-associated clearance requires selective pathway interruption, rescue that restores pathway function, and comparable initial drug injury.

Primary reports support the mechanistic connections; reviews provide structural context. Figures 3–4 present research proposals. All artwork is illustrative and does not represent patient measurements, observed staining results, quantitative organelle data or histological images.

Abbreviations: p62, sequestosome 1 protein; PB1, Phox and Bem1 protein-interaction domain; UBA, ubiquitin-associated domain; LC3, microtubule-associated protein 1 light chain 3; ATG8, autophagy-related protein 8; KEAP1, Kelch-like ECH-associated protein 1; NRF2, nuclear factor erythroid 2-related factor 2; IL-1, interleukin 1; TRAF6, tumor necrosis factor receptor-associated factor 6; aPKC, atypical protein kinase C; IKK, IκB kinase (IκB, inhibitor of nuclear factor κB); NF-κB, nuclear factor kappa-light-chain-enhancer of activated B cells; mTORC1, mechanistic target of rapamycin complex 1; Raptor, regulatory-associated protein of mechanistic target of rapamycin complex 1; Rag, Ras-related guanosine triphosphate-binding proteins; TNBC, triple-negative breast cancer; pCR, pathological complete response; RCB, residual cancer burden.

Supplementary Table S3. Evidence counts by type for selected claims in this review.

Claim and selected reports	Human histology/IHC	Human genomic/RNA	Patient-derived model	Other experimental model	Interpretation
p62 and clinical outcome in TNBC-focused cohorts [17,18]	2	0	0	0	The cohorts reported different directions of prognostic association; neither study demonstrated mitophagy.
p62 assessment before and after neoadjuvant therapy in a mixed breast cancer cohort [19]	1	0	0	1	The clinical tissue observations and cell experiments come from one publication. The reported baseline and residual p62 category counts have different denominators and do not establish within-patient change.
Autophagy-marker expression by cellular compartment in mixed breast cancer [34]	1	0	0	0	The mixed breast-cancer cohort documents subtype- and compartment-dependent staining, providing context for the TNBC-focused findings in [18].
Compartment-specific associations between p62 and COX-2 [35]	1 [35]	0	1 [35]	0	The same publication includes temporal clinical cohorts of 218 and 221 cases and a separate analysis of 37 PDX tumors. These static associations do not measure mitochondrial turnover or validate a composite profile.
Clinical NRF2 observations and experimental redox intervention [36,37,39]	3 [36,37,39]	2 [36,39]	1 [39]	1 [39]	Taddei et al. report descriptive MET/NRF2 IHC in TNBC biopsy specimens, public expression analyses and experiments in PDO and cellular models. These assays and models provide different evidence components and do not independently validate a composite profile.
A hypoxia/autophagy marker panel in pretreatment tissue [38]	1	0	0	0	The study reports human pathology associations but does not measure p62 or mitochondrial turnover.
Mitochondrial adaptation and survival in the chemotherapy setting [40,45,51,63,67]	1 [40]	2 [40,45]	5 [40,45,51, 63,67]	4 [40,45,63,67]	Andrzejewski et al. include PROMIX RNA analyses, paired BCL-XL IHC and experiments in the HCI-002 PDO. The human data do not measure mitochondrial flux or establish a mitochondrial dependency. Different components of one publication do not constitute independent clinical validation.
A BNIP3-specific mammary mechanism and human associations [29]	0	1	0	1	The same publication includes mouse experiments and analyses of human datasets; it does not validate BNIP3 IHC in human tissue.
The TNC-related immune mechanism [44]	1	1	0	1	The human correlation and RNA analyses, together with causal experiments in models, concern non-mitochondrial cargo.
TNFRSF19 clinical associations and a mitophagy mechanism investigated in models [41]	1 [41]	1 [41]	0	1 [41]	The publication includes institutional IHC/methylation data, public expression analyses and conventional model experiments. The 42 TCGA cases are a subset of the 113. These components do not provide independent human validation of mitophagy or the proposed profiles.
Validation of any complete profile proposed in Table 3	0	0	0	0	No selected study independently validated a complete profile proposed in Table 3 or a corresponding model-to-tissue classifier.

The counts refer to the named, selected publications that contribute each listed type of evidence. A publication is counted once per cell, even when it includes several datasets or experiments. The same paper may appear in more than one column or row; these repeated appearances do not, by themselves, establish independent replication. Counts are not summed to derive patient totals, numbers of independent cohorts or a denominator for the entire literature. Reanalysis of public expression data does not represent new patient recruitment.

Abbreviations: IHC, immunohistochemistry; RNA, ribonucleic acid; p62, sequestosome 1 protein; TNBC, triple-negative breast cancer; COX-2, cyclooxygenase 2; PDX, patient-derived xenograft; NRF2, nuclear factor erythroid 2-related factor 2; MET, MET proto-oncogene receptor tyrosine kinase; PDO, patient-derived organoid; BCL-XL, B-cell lymphoma-extra-large protein; BNIP3, B-cell lymphoma 2-interacting protein 3; TNC, tenascin-C; TNFRSF19, tumor necrosis factor receptor superfamily member 19; TCGA, The Cancer Genome Atlas. PROMIX is the trial/dataset name and HCI-002 is a patient-derived model identifier.
